# Putative glioblastoma origin-like cells in the subventricular zone: isolation and characterization

**DOI:** 10.1038/s12276-026-01801-4

**Published:** 2026-08-06

**Authors:** Hyeong-Cheol Oh, Ran Joo Choi, Se-Young Jo, Eunchae Yeo, Euna Jo, Jin-Kyoung Shim, Kibyeong Kim, Seo Jin Kim, Hye Joung Cho, Hyun Jung Kim, Joo Ho Lee, Seon-Jin Yoon, Ryong Nam Kim, Jeongsoo Won, Jiho Park, Seunghyun Kang, Jihwan Yoo, Ju-Hyung Moon, Tae Hoon Roh, Eui-Hyun Kim, Se Hoon Kim, Jong Hee Chang, Albert H. Kim, Hyun Seok Kim, Jeong Ho Lee, Hoon Kim, Sangwoo Kim, Seok-Gu Kang

**Affiliations:** 1 Department of Neurosurgery, Brain Tumor Center, Severance Hospital, Yonsei University College of Medicine, Seoul, Republic of Korea; 2 Brain Tumor Translational Research Laboratory, Department of Biomedical Sciences, Yonsei University College of Medicine, Seoul, Republic of Korea; 3 Department of Pharmacology, Yonsei University College of Medicine, Seoul, Republic of Korea; 4 Brain Research Institute, Yonsei University College of Medicine, Seoul, Republic of Korea; 5 Graduate School of Medical Science, Brain Korea 21 Plus Project for Medical Sciences, Yonsei University College of Medicine, Seoul, Republic of Korea; 6 Department of Biomedical Systems Informatics, Yonsei University College of Medicine, Seoul, Republic of Korea; 7 Department of Biohealth Regulatory Science, School of Pharmacy, Sungkyunkwan University, Suwon, Republic of Korea; 8 Department of Anatomy, Korea University College of Medicine, Seoul, Republic of Korea; 9 Department of Radiation Oncology, Seoul National University Hospital, Seoul National University College of Medicine, Seoul, Republic of Korea; 10 Department of Biopharmaceutical Convergence, School of Pharmacy, Sungkyunkwan University, Suwon, Republic of Korea; 11 Department of Neurosurgery, Gangnam Severance Hospital, Yonsei University College of Medicine, Seoul, Republic of Korea; 12 Department of Pathology, Severance Hospital, Yonsei University College of Medicine, Seoul, Republic of Korea; 13 Department of Neurosurgery, Washington University School of Medicine, St. Louis, MO USA; 14 The Brain Tumor Center, Siteman Cancer Center, Washington University School of Medicine St. Louis, St. Louis, MO USA; 15 Department of Biomedical Sciences, Yonsei University College of Medicine, Seoul, Republic of Korea; 16 Graduate School of Medical Science and Engineering, Korea Advanced Institute of Science and Technology (KAIST), Daejeon, Republic of Korea; 17 Postech Biotech Center, Pohang University of Science and Technology (POSTECH), Pohang, Republic of Korea; 18 Department of Neurosurgery, Graduate School of Medical Science, Brain Korea 21 Project, Yonsei University College of Medicine, Seoul, Republic of Korea

**Keywords:** CNS cancer, Cancer genomics, Cancer stem cells, Stem-cell research, Cancer in the nervous system

## Abstract

Glioblastoma (GBM) remains lethal despite maximal therapy. The adult subventricular zone (SVZ), a neural stem-cell niche, has been implicated as a potential site of origin, yet the identity and functional properties of putative GBM origin-like cells (GBM-OCs) within the SVZ remain unclear. An SVZ-restricted somatic mutation mouse model (Cre-induced EGFRvIII expression with Trp53 and Pten disruption) was established and mouse SVZ-derived cells were prospectively isolated for functional and molecular profiling. Self-renewal, multipotency, invasive potential and tumour-initiating capacity were assessed relative to control SVZ cells and matched tumour-derived tumourspheres. Whole-genome and RNA sequencing defined genomic and transcriptional alterations during early progression. Mouse GBM-OCs exhibited self-renewal and multilineage differentiation and initiated tumours only after re-implantation into the SVZ (11/29, 38%), whereas direct striatal implantation failed (0/25, 0%), indicating context-dependent tumorigenic potential associated with the SVZ microenvironment. In contrast, tumour-derived tumourspheres retained tumorigenic capacity upon implantation into both the SVZ and the striatum. During progression from mouse GBM-OCs to tumours, whole-chromosome and arm-level aneuploidies accumulated. In patients with GBM, multi-region single-nucleus RNA sequencing of tumour-free SVZ, matched tumours and tumour-free cortex identified rare neural stem cell-like, astrocyte-like and oligodendrocyte precursor-like SVZ populations transcriptionally aligned with GBM programmes. These cells showed single-nucleus RNA-inferred chromosome 7 gain and/or chromosome 10 loss signals, with concordant low-frequency copy-number alterations in the SVZ detected by exome sequencing and enriched in matched tumours. Together, these findings support the presence of SVZ-resident stem or progenitor-like populations with early GBM-associated features, consistent with putative GBM-OCs, and highlight the SVZ niche as a potential target for early detection and niche-informed therapeutic strategies.

## Isolating glioblastoma origin cells from the subventricular zone

Glioblastoma (GBM) remains a challenging cancer with a poor prognosis. GBM often originates from the subventricular zone (SVZ), a neural stem-cell niche. This study explores the potential of SVZ-derived cells as GBM origin-like cells (GBM-OCs), using a mouse model with SVZ-restricted somatic mutations. Researchers isolated and characterized these cells, assessing self-renewal, differentiation and tumour-initiating capacities. These cells, termed mouse GBM-OCs, exhibit context-dependent tumorigenic potential, forming tumours only when re-implanted into the SVZ. Whole-genome sequencing of the mouse model revealed progressive accumulation of copy-number alterations during progression from mouse GBM-OCs to tumours. Human GBM samples contained similar SVZ-resident populations with early GBM-associated transcriptional programmes and chromosomal alterations (chromosome 7 gain and/or chromosome 10 loss). These findings suggest that SVZ-resident stem or progenitor-like populations may represent early GBM-OCs, highlighting the SVZ as a potential target for early detection and therapeutic strategies.

This summary was initially drafted using artificial intelligence, then revised and fact-checked by the author.

## Introduction

Glioblastoma (GBM) is characterized by a dismal prognosis, with a median overall survival of 14.6–20.8 months despite aggressive treatment, including surgical resection, chemotherapy and radiation therapy^1,2^. Numerous therapeutic trials have sought to improve patient survival^3–9^, yet major advances remain elusive, largely due to the high rate of recurrence^10^. As a result, standard therapy still relies heavily on radiation therapy and the alkylating agent temozolomide. A deeper understanding of the mechanisms underlying GBM initiation, progression and recurrence is essential to overcoming therapeutic limitations. Supporting the importance of early tumorigenic events, large-scale evolutionary reconstructions of GBM cohorts have shown that copy-number alterations (CNAs), particularly chromosome 7 gain and/or chromosome 10 loss, represent early, clonally dominant events that can arise years before clinical diagnosis^11,12^. These observations, together with recent insights into early mutation-bearing precursor populations and their cellular context in glioma development^13,14^, suggest that defining the earliest GBM-primed states and their cellular context may be critical for improving recurrence control.

Recent studies of GBM pathogenesis have suggested the existence of putative GBM origin-like cells (GBM-OCs) residing in the subventricular zone (SVZ) that may acquire GBM-associated alterations in the SVZ and subsequently contribute to tumour development in distant cortical regions^15,16^. In this framework, ongoing migration of GBM-OCs from the SVZ effectively explains distant recurrence even after supratotal resection^10,16,17^, as demonstrated in an in vivo SVZ somatic mutation model generated by electroporation (SVZ-mutation model)^15,17^. However, direct isolation and molecular characterization of putative GBM-OCs have not been achieved, limiting mechanistic studies of early GBM evolution and the evaluation of therapeutic strategies. Here, the term ‘putative GBM origin-like cells’ is used operationally to refer to SVZ-derived stem or progenitor-like cells that harbour GBM-associated alterations and exhibit context-dependent tumorigenic potential.

This study tested the hypothesis that SVZ-derived stem or progenitor-like cells harbour intrinsic tumorigenic potential in an SVZ-associated microenvironmental context and acquire characteristic CNAs during tumorigenesis. Using an SVZ-restricted somatic mutation mouse model, putative mouse GBM origin-like cells (mGBM-OCs) were isolated and their cellular and molecular features relevant to GBM pathogenesis were systematically characterized. The key properties, including self-renewal, differentiation and tumorigenic potential, were compared with those of control SVZ cells and mouse GBM tumourspheres (mGBM-TSs) derived from primary tumours in the same model. Integrated whole-genome sequencing (WGS) and bulk RNA sequencing (RNA-seq) were used to profile the genomic and transcriptomic landscape, CNAs, and pathway alterations of mGBM-OCs. Finally, by applying multi-region single-nucleus RNA sequencing (snRNA-seq) and deep whole-exome sequencing (WES) to paired, neuropathology-confirmed tumour-free SVZ and tumour samples from patients with GBM, whether putative GBM-OC populations exhibiting early chromosomal alteration signals could be detected in the tumour-free human SVZ was assessed.

## Materials and methods

### Ethics

This study was approved by the Institutional Review Board of Severance Hospital for transcriptomic analysis of human samples (IRB 4-2021-1319). The study procedures adhered to the tenets of the 1964 Declaration of Helsinki. All patients provided written informed consent for participation in the study and for publication. All animal experiments were approved by the Yonsei University College of Medicine Institutional Animal Care and Use Committee (LML-13-276).

### Cell culture

The tumour or the SVZ specimen was dissected from SVZ somatic mutation model mice using a stereomicroscope (S9i, Leica)^18^. Subsequent cell isolation from the specimens was conducted as previously described^19^ (Supplementary Video 1). Cell suspensions were cultured in complete medium composed of DMEM/F-12 containing B27 supplements (1X; Invitrogen), 20 ng/ml of basic fibroblast growth factor (bFGF; Novoprotein), 20 ng/ml of epidermal growth factor (EGF; Novoprotein), and 1% penicillin–streptomycin (15140122, Thermo Fisher). All in vitro experiments were performed under the above culture conditions using early-passage cells (passages <20).

### Direct cell counting

Dissociated cells were plated in triplicate in six-well plates at a density of 1.0 × 10^5^ cells/well in complete medium. At the indicated time points, cells were harvested and counted manually using a haemocytometer under a light microscope. Cell viability was determined by trypan blue exclusion, and total live cell numbers were averaged across replicates.

### Sphere formation assay

Cells were plated as a single-cell suspension in 96-well plates and cultured for 11 days. The number of sphere-positive wells was counted, and the proportion of sphere-positive wells in each group was calculated and presented as a percentage. Images of sphere-positive wells were captured and analysed using ToupView software (ToupTek Photonics). Cells were plated at 5 cells/well (single-cell suspension) in 60 wells per condition. A well was considered sphere-positive if it contained ≥1 sphere with a diameter ≥50 µm on day 11.

### 3D invasion assay

The invasiveness of control SVZ cells, mGBM-OCs and mGBM-TSs was determined using a 3D invasion assay. Single dissociated cells (3,000 cells) were seeded into each well of a PrimeSurface 96M plate (Shimadzu). After 24 h, each well was filled with a Matrigel matrix composed of Matrigel, collagen type I (Corning) and complete medium prepared at a Matrigel-to-collagen I-to-medium ratio of 55:120:325 (final collagen I concentration, 0.72 mg/ml). After 30 min, complete medium (30 μl/well) was added to the gel matrix to avoid drying. Images were captured and the invasive area was analysed using an Operetta CLS (PerkinElmer). The invasive area was quantified as (area at 72 h − area at 0 h)/area at 0 h for each well using brightfield or fluorescence images, and values were averaged across replicates. Brightfield images were acquired for all groups at 0 and 72 h. For tdTomato-expressing cells, additional fluorescence images were acquired using identical imaging settings.

### Neuroglial differentiation

Neuroglial differentiation in control SVZ cells, mGBM-OCs and mGBM-TSs was induced by replacing the culture medium with DMEM/F-12 supplemented with B27 (1X; Invitrogen), 10% fetal bovine serum (Invitrogen) and 1% penicillin–streptomycin, followed by incubation on a Corning 4-well chamber slide for 2 weeks. Spheres were seeded on chamber slides (C6932, MERCK) prior to differentiation. Differentiation toward neuronal and glial lineages was assessed by immunostaining for the neuronal marker TUBB3 (Santa Cruz) and the astrocytic marker GFAP (Santa Cruz), respectively.

### Animals

Mice with LSL-tdTomato (C57BL/6 strain, The Jackson Laboratory) were bred with mice carrying the LSL-EGFRvIII alleles (FVB strain), and neonatal pups (P1) underwent in vivo electroporation of the SVZ with plasmid solution. The plasmid mixture included Cre recombinase and Cas9 together with single-guide RNAs targeting Trp53 and Pten (final total DNA concentration >2.5 μg/μl) with 1% Fast Green, as previously reported^15^. Both male and female pups were used unless otherwise indicated.

No IDH1 or IDH2 alterations were introduced in this model. As tumorigenesis was initiated through SVZ-targeted induction of Trp53 and Pten loss together with EGFRvIII activation, the model is genetically defined and intended to recapitulate key molecular features of IDH-wild-type GBM; therefore, no IDH-based exclusion criteria were applied. All mouse cohorts, harvested tissues and sequencing datasets presented in this study were newly generated for the current work. Although the mouse model builds on the previously reported SVZ-targeted electroporation or genome-editing framework^15^, no mouse specimens or datasets were reused from the prior publication.

### Orthotopic engraftment

For the orthotopic allograft model, male 6–8-week-old athymic nude mice (Central Lab Animal Inc.) were housed in micro-isolator cages under sterile conditions and monitored for at least 1 week before the experiments to ensure proper health. Mice were anaesthetized with Zoletil (30 mg/kg; Virbac Korea) and xylazine (10 mg/kg; Bayer Korea) administered intraperitoneally. Dissociated cells (2 × 10^5^) were orthotopically implanted (day 0), as previously described^20,21^. IVIS fluorescence imaging (tdTomato) was performed using an in vivo imaging system, and images were analysed using Living Image v4.2 software (Caliper Life Sciences).

For the SVZ injection model, 7-week-old athymic nude mice (Central Lab Animal Inc.) were used for all experiments. The bregma was identified and used as the reference point for stereotaxic coordinates. For SVZ injections, the following coordinates were used relative to bregma: anterior–posterior –0.2 mm, medio-lateral –1.05 mm and dorso-ventral –1.7 mm from the dura. A small hole was drilled in the skull at the predetermined coordinates using a micro-drill. A 10-μl Hamilton syringe was mounted onto the stereotaxic arm (RWD Life Science) and lowered slowly to the target dorso-ventral coordinate. The cell suspension (2 × 10^5^) was delivered at a rate of 0.5 μl/min, with a total volume of 3 μl. Following the injection, the needle was left in place for 5 min to minimize backflow before being slowly withdrawn from the brain. The scalp incision was closed using 6-0 sterile sutures, and a topical antibiotic ointment was applied to the wound area. The injection site was validated by coronal sectioning with ventricular and SVZ landmarks as well as tdTomato fluorescence (or dye track) at 48 h post-injection and at endpoint. Tumour take was defined as the presence of histologically confirmed tdTomato-positive tumour cells within the injected hemisphere and/or imaging-positive lesions, assessed after 7 weeks post-injection.

### Image analysis of mouse brain

Mouse brains were obtained, fixed in 10% formalin, cryoprotected overnight in 30% buffered sucrose and stored in frozen blocks in OCT (optimal cutting temperature compound) at −80 °C. Coronal cryostat-cut sections (10-μm thick) were obtained at coronal planes corresponding to AP +1, −1, −2 and −3 mm relative to bregma. A mounting medium containing 4′,6-diamidino-2-phenylindole (DAPI; H-1200-10, Vector Laboratories) was used for nuclear staining. Images were obtained using a Zeiss LSM700 confocal microscope (Zeiss). For the haematoxylin and eosin staining, 4 μm cryostat-cut sections were used.

### Mouse and human RNA-seq analysis

Detailed methodologies for mouse bulk RNA-seq analysis, mouse cell preparation and single-cell RNA-seq analysis, single-nucleus isolation of human samples and library preparation for snRNA-seq, as well as human bulk RNA-seq-based gene expression and CNA analyses, are provided in the Supplementary information (Supplementary Methods).

### Mouse WGS and bulk RNA-seq data generation and pre-processing

The WGS libraries were prepared using the TruSeq Nano DNA Sample Prep Kit, whereas RNA-seq libraries were prepared using the TruSeq Stranded mRNA Sample Prep Kit. Sequenced raw reads from WGS were mapped against the GRCm38 mouse genome reference using the Burrows–Wheeler Aligner (BWA-MEM, v0.7.17)^22^, followed by MarkDuplicates and FixMateInformation using GATK (v4.1.9.0). Similarly, sequenced raw reads from RNA-seq were mapped against GRCm39 with GENCODE M35 annotation using the STAR aligner (v2.7.10a)^23^, followed by MarkDuplicates using GATK (v4.1.9.0). WGS libraries were generated to an average depth of approximately 60× per sample. For the bulk RNA-seq data, approximately 30 million paired-end reads were generated per sample, of which ~70% were retained as non-duplicate read pairs based on duplicate marking.

### Allele-specific CNA analysis of mouse bulk WGS data

Allele-specific copy-number status for all samples was inferred using Sequenza (v2.1.2)^24^ on the WGS data. The BAM file for each case sample (either mGBM-OC or mGBM-TS) was input into Sequenza along with the corresponding normal blood sample BAM file. Normal blood was collected from the same mouse and processed as matched normal. Sequenza calculates the relative depth, which refers to the comparison of sequencing coverage between the case sample and the corresponding normal sample across the genome. Additionally, Sequenza measures B-allele frequencies at heterozygous single-nucleotide polymorphism sites within the genomic region. By calculating the maximum probability, the tool infers the allele-specific copy number for the given genomic region. To evaluate the enrichment of chromosomal alterations during tumour progression, odds ratios and adjusted *P* values (Fisher’s exact test with Benjamini–Hochberg method) were calculated by comparing the frequency of copy-number gains or losses in mGBM-TS samples relative to mGBM-OC samples for each chromosome.

### Patient information

Bulk RNA-seq samples from 223 patients and snRNA-seq samples from 15 patients were collected, with all data obtained under written informed consent at Severance Hospital. Since July 2017, a targeted next-generation sequencing panel has been routinely performed for patients diagnosed with GBM^25^, with diagnoses and molecular classifications aligned with the WHO 2021 classification of tumours of the central nervous system. For patients diagnosed prior to July 2017, immunohistochemistry was conducted, and the results were recorded as percentage positivity for each marker (for example, IDH1 R132H, ATRX, p53 and others according to clinical practice at the time). All GBM diagnoses were reviewed and aligned to the WHO 2021 criteria and IDH-wild-type status was confirmed by targeted sequencing when available and/or by IDH1 R132H immunohistochemistry (with additional IDH1 and IDH2 sequencing as clinically indicated). Cases meeting criteria for IDH-mutant astrocytoma or oligodendroglioma (WHO 2021) were excluded.

### MRI

Perioperative axial or coronal MRI was used for preoperative planning and to visualize the SVZ biopsy sites. The planned SVZ removal area was identified by the surgeons, and the thin layer was dissected in the grossly non-invaded area. SVZ tissue was subsequently confirmed as tumour-free by neuropathology (criteria described in Supplementary Methods).

### Library preparation for snRNA-seq

Sequencing was performed at Yonsei University Genome Center or Macrogen using a 10× Chromium Controller (10× Genomics) according to the manufacturer’s protocol (10× Genomics) (see Supplementary Methods for details).

### Processing human snRNA-seq data

The Cell Ranger pipeline (v7.2.0) was used to process the raw FASTQ data and generate gene count matrices. The Cell Ranger count command was used with the default parameter settings for the data from 12 patients (GN1, GN2, GN3, GN5, GN6, GN7, GN8, GN20, GC14, CN9, CN10 and CN11). For four patients (GN15, GN16, GN17, GN18), whose snRNA-seq data were generated with snATAC, only the snRNA data were used, and the --chemistry=ARC-v1 option was added. The reference genome was downloaded from the 10× Genomics website (‘refdata-gex-GRCh38-2020-A’).

Genes detected in at least three cells were retained. Cells with a minimum of 500 and a maximum of 10,000 detected genes, at least 1,000 unique molecular identifiers, and less than 10% mitochondrial reads were kept for downstream analysis. Doublet Finder (v2.0.4)^26^ was used to remove doublets. The pK value was selected based on the maximum value in the parameter sweep, and the multiplet rate was determined by referring to the 10× Genomics website.

The processed data were used as input to perform batch correction using the Harmony^27^ method with the IntegrateLayers command in the Seurat (v5.0.1) package for samples from the same disease context (tumour-free SVZ, primary GBM, recurrent GBM, tumour-free cortex and tumour-infiltrated SVZ for patients with GBM or control SVZ and control cortex from patients with non-glial tumours). If an SVZ sample was pathologically tumour free but the cell cluster or CNA patterns strongly suggested tumour infiltration (for example, tumour-like cluster proportions and concordant large-scale CNA profiles), it was classified as tumour-infiltrated SVZ (GN20). After integration, normalization, scaling and linear dimension reduction were performed with default parameters. The dimensionality of the dataset was determined using the ElbowPlot, with the chosen dimensions (nPC) being 30. FindNeighbors and FindClusters were performed sequentially, using the selected nPC values and a resolution of 0.5. The uniform manifold approximation and projection algorithm was used to visualize the dataset.

### Human single-cell classification

To annotate the cell cluster types, the marker genes for each cluster were identified using FindAllMarkers from the Seurat package. Reference-based annotation was then performed by utilizing multiple established methods, including ScType^28^, EasyCellType^29^ and CellMarker (v2.0)^30^, along with curated cell-type databases available in GBmap^31^, Neftel et al.^32^ and PanglaoDB^33^. To validate whether the marker genes were highly expressed in the corresponding clusters, feature and dot plots were generated using the Seurat package.

### Two-dimensional representation of malignant cellular states

To assess metamodule scores, the AddModuleScore function was used in the Seurat package with the metamodule gene list from Neftel et al. for clusters highly expressing EGFR and PTPRZ1, including astrocyte (AC)-like, neural stem cell (NSC)-like, oligodendrocyte precursor (OPC)-like, mesenchymal (MES)-like, cycling, and angiogenic clusters. Relative metamodule scores were calculated following Neftel’s approach. Cells were first classified into OPC-like and neural progenitor cell (NPC)-like versus AC-like or MES-like using the metric D, where D = max(score of OPC-like, NPC-like) – max(score of AC-like, MES-like). This value defined the y-axis for all cells. Next, for OPC-like and NPC-like cells (D >0), the x-axis was determined by the difference between OPC-like and NPC-like scores, whereas for AC-like and MES-like cells (D <0), the x-axis was determined by the difference between AC-like and MES-like scores.

### Inferring CNVs from human snRNA-seq data

To predict CNAs and cluster cells by similar CNA profiles, inferCNV (v1.18.1) was applied to the GBM and non-glial tumour datasets. In the CNV analysis, genes were selected with a 0.1 cut-off. Sub-clustering was conducted using the Leiden partitioning method, and CNV prediction was based on the Hidden Markov Model. Oligodendrocytes and microglia from GBM and non-glial tumour datasets were consistently used as non-malignant reference cells. The genomic position file (‘hg38_gencode_v27.txt’) was downloaded from the TrinityCTAT website.

For visualizing clusters exhibiting chromosome 7 gains or chromosome 10 losses in GBM or non-glial tumour datasets, a 0.5 threshold was applied to several parameters automatically calculated by the inferCNV package such as proportion_scaled_dupli_chr7_p (or q arm) and proportion_scaled_loss_chr10_p (or q arm). These parameters indicate the threshold for defining significant gains or losses, respectively. For a more conservative assessment, the presence of chromosome 7 gains or chromosome 10 losses was defined as occurring only if at least five cells exhibited the corresponding feature.

### Human WES analysis

A subset of patients in the current cohort (GBM-002 and GBM-008; corresponding to GN2 and GN3 in the snRNA-seq dataset) overlaps with those included in the prior study^15^. A subset of the human WES data used in this study was derived from previously published datasets (SRP145073)^15^ and was re-analysed here for comparative genomic analyses. In contrast, all multi-regional human snRNA-seq specimens and datasets were newly generated for the current work. Additional matched blood WES data were newly generated for GBM-001 and GBM-009 to improve somatic variant calling accuracy. No figure panels were reused from the prior publication. WES FASTQ raw data were aligned to the GRCh37 reference genome (‘human_g1k_v37_decoy’) using the Burrows–Wheeler Aligner^34^. Data preprocessing followed the GATK Best Practices and was performed using the GATK pipeline (v4.1.8.1). To ensure that the samples originated from the same patient, fingerprinting was performed with NGSCheckMate (v1.0.1)^35^, which compared each sample against all others. Exome capture was performed using the Agilent Human All Exon 50 Mb kit (V5) according to the manufacturer’s instructions.

Copy-number segmentation was performed following GATK Best Practices, using GBM, SVZ and patient-matched normal (cortex or blood) samples. When both cortex and blood samples were available, the blood sample was used as the matched normal control. Tumour purity and ploidy were calculated using TITAN (v1.24.0)^36^, which was provided with the denoised read counts and tumour allelic counts generated by the GATK Best Practice workflow. CNVs were represented as log2 ratios, visualized using IGV (v2.12.3)^37^.

Somatic mutations were identified in GBM and SVZ samples, with patient-matched blood as the control, using Mutect2 (GATK v4.1.8.1) following GATK Best Practices. The identified somatic mutations were normalized using bcftools (v1.10.1), annotated with Funcotator (v4.1.0.0), and filtered to retain only sites with coverage greater than 100× in both SVZ and GBM. To eliminate potential artefacts while ensuring high-confidence mutations, only mutations detected in GBM with a variant allele frequency ≥0.1 and FILTER = PASS were included in the final analysis.

### Statistical analysis

Each biological replicate represents cells isolated from an independent mouse. For in vitro functional assays, technical wells were averaged within each independent mouse-derived cell culture or cell line before group-level statistical testing and were not treated as independent biological replicates. For human analyses, each biological replicate corresponds to an independent patient sample. Replicate numbers for each group are indicated in the figure legends (for example, control SVZ, *n* = 3; mGBM-OCs, *n* = 3; mGBM-TSs, *n* = 6 for bulk RNA-seq). Bulk RNA-seq differential expression analysis was performed using the DESeq2 Wald test, and differentially expressed genes (DEGs) were defined as |log2 fold-change|>1 with Benjamini–Hochberg adjusted *P* <0.05 unless otherwise specified. Marker genes for single-cell RNA-seq and snRNA-seq clusters were identified using the Wilcoxon rank-sum test implemented in Seurat FindAllMarkers. Gene set enrichment analysis was performed using the fgsea package with MSigDB hallmark gene sets, and an adjusted *P* <0.05 was considered significant. Human Gene Ontology enrichment analyses were performed using clusterProfiler as described in the Supplementary Methods. The statistical significance of DEG overlap between mGBM-OCs and mGBM-TSs was assessed using a one-sided hypergeometric test, using 6,308 genes passing DESeq2 independent filtering as the background set. Benjamini–Hochberg correction for multiple testing was applied in all genome-wide analyses, including DEG analysis, pathway enrichment and chromosome-level copy number comparisons, with chromosome-level copy number enrichment assessed using Fisher’s exact test followed by Benjamini–Hochberg correction. Survival curves were estimated using the Kaplan–Meier method, and group comparisons were evaluated using log-rank tests where applicable. For quantitative assays (cell counting, sphere formation, invasion), Kruskal–Wallis tests with Dunn’s post hoc test were performed using Python. Unless otherwise specified, a two-sided *P* <0.05 was considered statistically significant.

### Data visualization

Figures were created using Python (3.9.12) and R (4.3.2). Schematics were created using BioRender.com.

## Results

### SVZ somatic mutation model enables isolation of mGBM-OCs

**Fig. 1:**
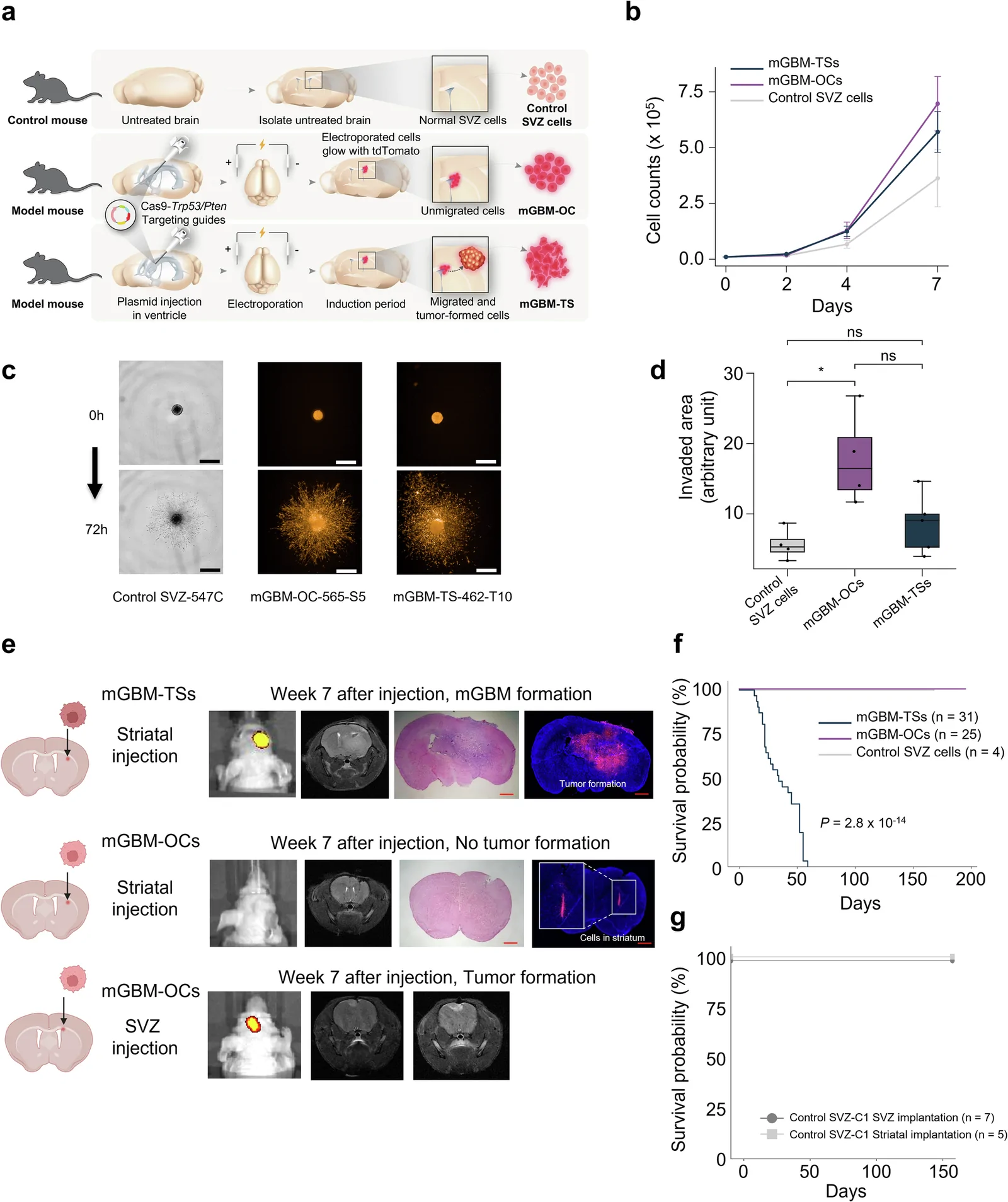
Characterization of putative mGBM-OCs and mGBM-TSs. **a**, Schematic overview of in vivo electroporation into the lateral ventricle adjacent to the subventricular zone (SVZ) and the subsequent isolation of three cell types. A plasmid encoding single-guide RNA (sgRNAs) targeting *Trp53* and *Pten*, together with Cas9 and Cre recombinase, was injected into the lateral ventricle of LSL-EGFRvIII;LSL-tdTomato mice. Cre recombinase induces EGFRvIII and tdTomato expression in targeted SVZ cells. Mouse glioblastoma origin-like cells (mGBM-OCs) were isolated from the SVZ in the SVZ somatic mutation model, mouse glioblastoma tumourspheres (mGBM-TSs) were isolated from the resulting tumours, and control SVZ cells from the SVZ of null mice. **b**, Comparison of cell proliferation across the three groups (mGBM-OCs, mGBM-TSs and control SVZ cells) at days 0, 2, 4 and 7. The number of biological replicates for mGBM-OCs, mGBM-TSs and control SVZ cells is *n* = 4, 3 and 3, respectively. Data points and error bars indicate the mean and standard deviation of independent biological replicates. **c**, Representative images from the 3D invasion assay acquired at 0 and 72 h after Matrigel matrix loading. Brightfield images were acquired for all groups; for tdTomato-expressing cells (mGBM-OCs and mGBM-TSs), corresponding fluorescence images are shown. **d**, Invaded area was quantified using a 3D invasion assay for the three groups. The control SVZ group included Control SVZ-C1, Control SVZ-546-C, Control SVZ-547-C and Control SVZ-548-C; the mGBM-OC group included mGBM-OC-S0, mGBM-OC-445-S20, mGBM-OC-565-S5 and mGBM-OC-586-S5; and the mGBM-TS group included mGBM-TS-T1, mGBM-TS-462-T10, mGBM-TS-475-T10, mGBM-TS-520-T10 and mGBM-TS-523-T10. Boxes represent the 25th, 50th (median), and 75th percentiles and whiskers extend to 1.5 times the IQR. A Kruskal–Wallis test was performed, showing a significant difference between mGBM-OCs and control SVZ cells. Statistical significance: * *P* <0.05. Scale bar: 500 μm. **e**, Schematic illustration of the implantation of mGBM-TSs and mGBM-OCs into the striatum or SVZ. Representative images from in vivo fluorescence imaging (tdTomato), MRI, histology and confocal microscopy are shown. **f**, Kaplan–Meier overall survival curves (time to death) for mGBM-TSs, mGBM-OCs and control SVZ cells, all implanted into the striatum. The number of mice used in each group is indicated by the respective ‘*n*’ value, with the full mGBM-OC striatal implantation cohort reported as 0 of 25 mice across repeated independent experiments. Mice were followed until death or a humane end point. **g**, Kaplan–Meier overall survival curves (time to death) for control SVZ cells (Control SVZ-C1) implanted in the SVZ and striatum, respectively.

We developed a mouse GBM model using in vivo SVZ electroporation^15^ of a plasmid containing single-guide RNAs targeting *Trp53* and *Pten*, together with Cas9 and Cre recombinase in LSL-EGFRvIII;LSL-tdTomato P1 mice (Fig. 1a and Supplementary Fig. 1a, b). Robust tdTomato positivity (marking Cre-recombined cells with EGFRvIII activation) was readily detected in the SVZ as expanding clones between 5 and 14 weeks post-electroporation. Induction of somatic mutations in these mice (*n* = 5) led to migration of tdTomato-positive cells from the SVZ to the cortex, resulting in tumour formation (Supplementary Fig. 1c). tdTomato-positive cells that remained confined to the SVZ (hereafter referred to as unmigrated cells) at 5, 14 and 20 weeks post-electroporation (Supplementary Video 1) were isolated and cultured, along with cells from the resulting cortical tumours and SVZ cells from mice in which tumours were not induced (control SVZ), both collected at 10–11 weeks post-electroporation. After culturing, both the unmigrated SVZ cells and tumour cells but not control SVZ cells remained tdTomato-positive and harboured indel mutations in *Trp53* and *Pten* (Supplementary Fig. 2a, b). On this basis, the cultured unmigrated tdTomato-positive SVZ cells were operationally defined as putative mGBM-OCs and the tumour-derived cells as mGBM-TSs (Supplementary Table 1).

### Cellular characterization of mouse control SVZ cells, mGBM-OCs and mGBM-TSs

Following cell isolation, whether these three types of cells (control SVZ cells, mGBM-OCs and mGBM-TSs) exhibit characteristics of NSCs was assessed. First, direct cell counting showed a trend toward increased proliferation in mGBM-OCs compared with control SVZ cells (*P* = 0.11) and a proliferative capacity comparable to mGBM-TSs (Fig. 1b). In neurosphere formation assays, control SVZ cells from mice in which tumours were not induced (control SVZ) formed neurospheres in an average of 64% of wells (*n* = 4), whereas mGBM-OCs and mGBM-TSs showed higher formation rates, with averages of 93% (*n* = 4) and 78% (*n* = 3), respectively. The differences in formation rates between groups were not statistically significant (*P* = 0.18 and *P* = 1.0, respectively) (Supplementary Fig. 2c). Moreover, when assessing lineage differentiation capacity, mGBM-OCs demonstrated the ability to differentiate into neuronal and glial lineages, similar to mGBM-TSs and control SVZ cells (Supplementary Fig. 2d). Collectively, these results indicate that mGBM-OCs possess self-renewal and differentiation abilities, consistent with a stem cell-like nature^38^. To further support their NSC-like identity at the molecular level, immunocytochemistry was performed, confirming expression of established neural stem or progenitor-associated markers, including SOX2 and CD133, in mGBM-OCs, as well as in control SVZ cells and mGBM-TSs (Supplementary Fig. 2e).

Next, the invasive characteristics of the three cell types were assessed. A 3D invasion assay showed that mGBM-OCs invaded a larger area than control SVZ cells, with a statistically significant difference (*P* = 0.025), and the invasion area of mGBM-OCs was comparable to that of mGBM-TSs (*P* = 0.23), demonstrating their invasive potential (Fig. 1c, d). Together with their ability to form tumours in vivo within the SVZ-mutation model, these findings support the migratory capacity of mGBM-OCs from their original location, as demonstrated by both in vitro and in vivo experiments.

### Tumour-initiating potential of mGBM-OCs varies depending on the implantation site

We investigated whether the tumorigenic potential of mGBM-OCs varies depending on the site of implantation. Direct striatal implantation of mGBM-OCs into athymic nude mice failed to generate tumours in an initial cohort (*n* = 10) (Fig. 1e), whereas mGBM-TSs implanted under identical conditions consistently formed tumours (Fig. 1e, f). Consistent with their fully transformed tumour phenotype, human GBM-TSs gave rise to tumours regardless of whether they were implanted into the striatum or the SVZ^39^ (Supplementary Fig. 3a). The inability of mGBM-OCs to initiate tumours was recapitulated in repeated experiments using cells from different mice, yielding no tumour formation across the full striatal implantation cohort (0 of 25 mice; mGBM-OC lines: S0, 445-S20, 565-S5, 586-S5; Fig. 1f and Supplementary Table 1). In contrast, when mGBM-OCs were implanted directly into the SVZ, 11 of 29 mice (38%) developed tumours in the cortex within 5–10 weeks after implantation (Supplementary Fig. 3b). Correct localization of the implantation site within the SVZ or ventricular wall region was confirmed by fluorescence microscopy at 48 h post-injection (Supplementary Fig. 3c) and at endpoint.

Control SVZ cells implanted in the same manner did not give rise to tumours following either striatal (0 of 5, 0%) or SVZ (0 of 7, 0%) implantation (Fig. 1g). Isolation of tumorspheres derived from SVZ-implanted mGBM-OCs and subsequent RNA-seq revealed differential gene expression enriched for oxidative phosphorylation (adjusted *P*= 1.7 × 10^–3^) and Myc target activation (adjusted *P* = 1.7 × 10^–3^), relative to control SVZ cells (Supplementary Fig. 4a, b), suggesting transcriptional reprogramming associated with tumorigenesis. Moreover, both tumorspheres derived from SVZ-implanted mGBM-OCs and mGBM-TSs exhibited similar gene set enrichment patterns (Supplementary Fig. 4a, b). Collectively, these findings indicate that the tumorigenic potential of mGBM-OCs varies depending on the implantation site and is context-dependent.

### Progressive CNAs from mGBM-OCs to mGBM-TSs

**Fig. 2:**
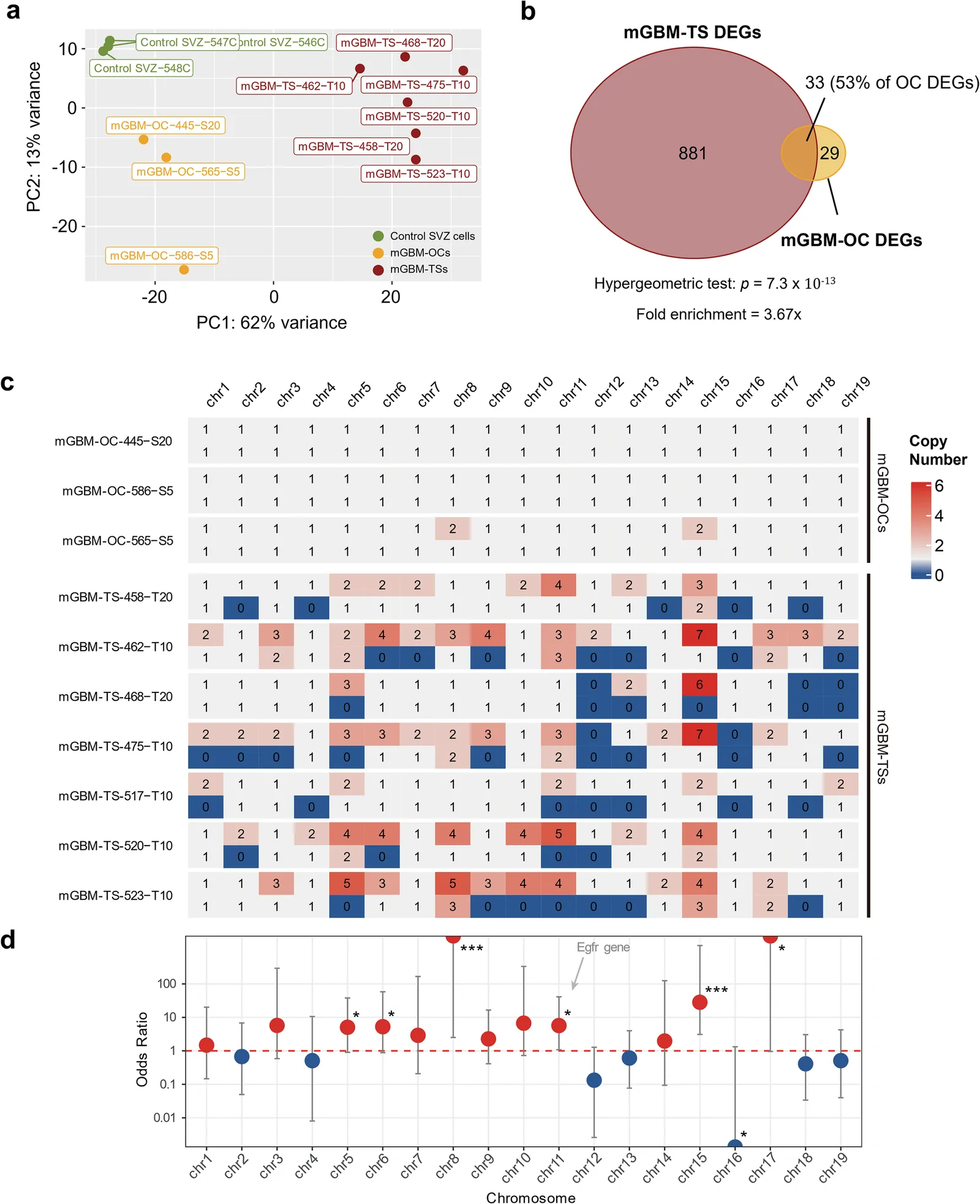
Progressive copy-number alterations from mGBM-OCs to mGBM-TSs. **a**, Principal component analysis plot showing gene expression patterns of the three groups: control subventricular zone (SVZ) cells, mouse glioblastoma origin-like cells (mGBM-OCs) and mouse glioblastoma tumourspheres (mGBM-TSs). Each point represents an independent biological replicate (control SVZ, *n* = 3; mGBM-OCs, *n* = 3; mGBM-TSs, *n* = 6); axis labels indicate the percentage of variance explained by each principal component (PC1, 62%; PC2, 13%). **b**, Differentially expressed genes (DEGs) were defined as |log2 fold-change|> 1 and Benjamini–Hochberg adjusted *P* <0.05 (DESeq2 Wald test). The number of DEGs specific to mGBM-OCs (orange), shared between mGBM-OCs and mGBM-TSs (overlap; 34 of 62 mGBM-OC DEGs, 54.8%), and specific to mGBM-TSs (dark red) are indicated. Statistical significance of the overlap was assessed using a one-sided hypergeometric test (*P* = 7.3 × 10^–13^; fold enrichment, 3.67), with the total number of measured genes with valid adjusted *P* values as the background (6,308 genes). **c**, Allele-specific absolute copy numbers per chromosome inferred from bulk whole-genome sequencing using Sequenza, relative to matched normal blood from the same mouse. mGBM-OCs (*n* = 3) and mGBM-TSs (*n* = 7) are shown. **d**, Odds ratios (OR) illustrating chromosome-specific copy-number alterations across all samples. Odds ratios were calculated for chromosome-level gains (red) or losses (blue) in mGBM-TSs relative to mGBM-OCs, with error bars indicating 95% confidence intervals and a dashed line indicating OR 1. Chromosome 11 gain includes the *Egfr* locus. Statistical significance: *adjusted *P* <0.05, ***adjusted *P* <0.001. Adjusted *P* values were obtained by multiple-testing correction across chromosomes (see Methods).

We characterized the molecular features of mGBM-OCs and compared them with those of mGBM-TSs to gain insight into changes associated with tumour development in this model. Bulk RNA-seq of control SVZ cells, mGBM-OCs and mGBM-TSs from 12 mice (3 biological replicates each for control SVZ cells and mGBM-OCs and 6 for mGBM-TSs) revealed distinct baseline transcriptomics profiles among these groups (Fig. 2a). Notably, mGBM-TSs exhibited a greater divergence from control SVZ cells, as reflected by the increased number of DEGs, whereas mGBM-OCs showed only a limited number of DEGs relative to control SVZ (Fig. 2b). Most DEGs observed in mGBM-OCs overlapped with those in mGBM-TSs (33 of 62, 53%), suggesting that transcriptional changes are already initiated in mGBM-OCs and become progressively amplified in mGBM-TSs (Supplementary Table 2). The full list of shared DEGs, including log2 fold-change values and adjusted *P* values for both comparisons, is provided in Supplementary Table 2. A one-sided hypergeometric test confirmed that this overlap was significantly greater than expected by chance (*P* = 7.3 × 10⁻¹³; fold enrichment, 3.67). Nonetheless, 29 DEGs were exclusive to mGBM-OCs and not shared with mGBM-TSs. These included Psph (log_2_FC = 6.08; serine biosynthesis)^40^, Plod2 (log_2_FC = 3.21; collagen cross-linking)^41,42^ and Psma5 (log_2_FC = 3.21; proteasome activity)^43,44^, which were transiently upregulated in mGBM-OCs but returned to near-baseline levels in mGBM-TSs (log₂FC = 0.30, 0.71 and 0.93, respectively; all adjusted *P* >0.35). This suggests that these metabolic and matrix remodelling-related changes represent early, transient responses to oncogenic mutations that are subsequently replaced by distinct transcriptional programmes during tumour progression (Supplementary Fig. 5a).

To further investigate genomic changes associated with tumour development in this model, WGS of mGBM-OCs and mGBM-TSs was performed. CNA analysis of these WGS data revealed widespread chromosomal gains and losses in mGBM-TSs, whereas mGBM-OCs displayed a relatively stable genomic landscape (Fig. 2c, d). A recurrent gain of chromosome 11, a region syntenic to human chromosome 7, was detected in mGBM-TSs (odds ratio 5.7, *P* = 0.022), consistent with de novo amplification of the *Egfr* locus^12^ (Supplementary Fig. 5b). Additionally, analysis of sex chromosomes revealed loss of the Y chromosome in two of three mGBM-OC samples and three of five mGBM-TS samples (Supplementary Fig. 5c). These findings are consistent with previously described oncogenic CNAs in human GBM^11,12,45^ and suggest that emerging chromosomal instability and specific copy-number changes may be associated with tumour progression in this model.

### Genomic and molecular characteristics of putative human GBM origin-like populations

**Fig. 3:**
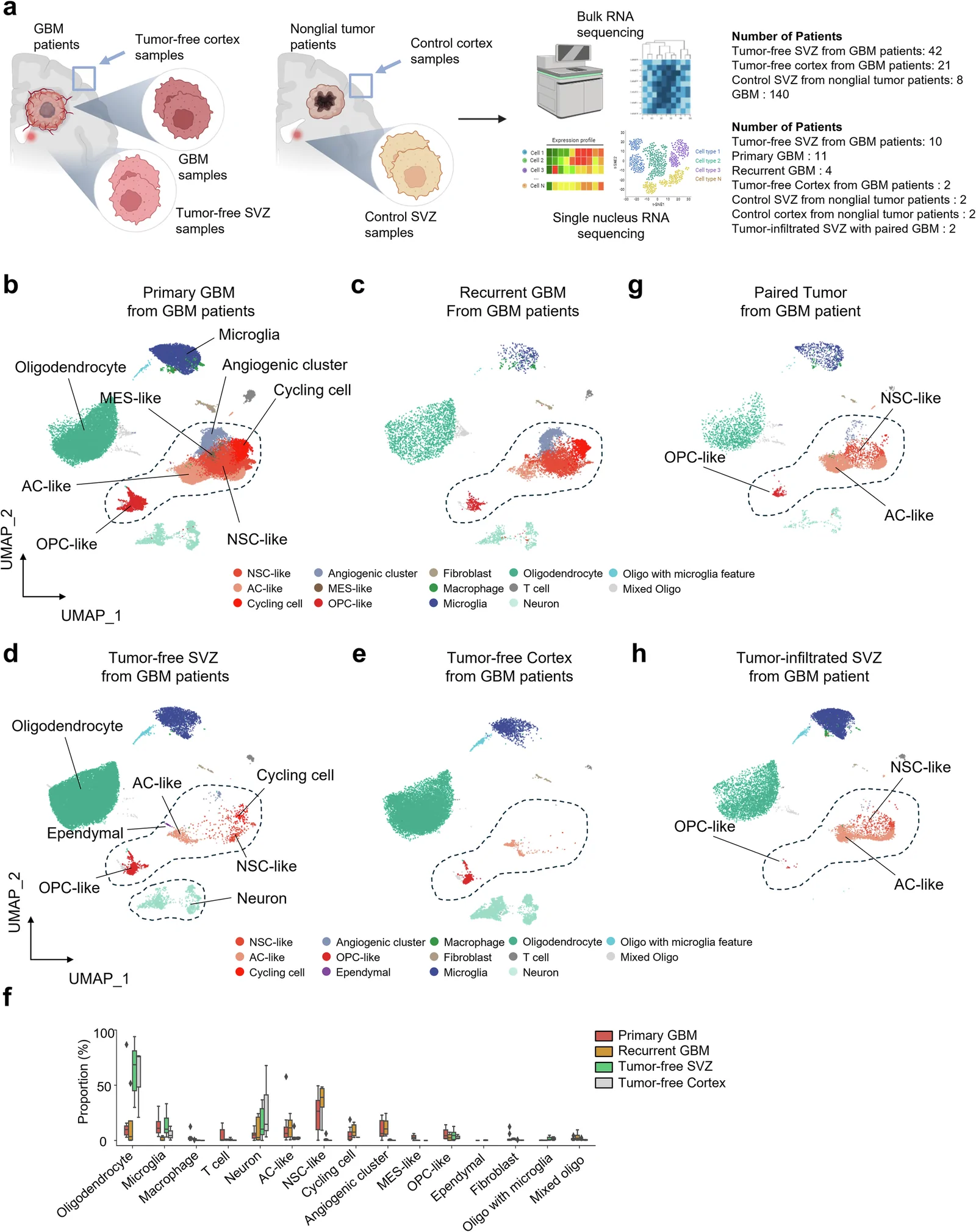
A single-nucleus landscape of human GBM and non-glial tumour samples. Schematic illustration depicting the sampling and RNA sequencing workflow for tumour-free subventricular zone (SVZ), primary glioblastoma (GBM), recurrent GBM and tumour-free cortex from patients with GBM, and control SVZ and control cortex samples from patients with non-glial tumours (part **a**). Bulk RNA sequencing and single-nucleus RNA sequencing (snRNA-seq) workflows are shown, with sample numbers indicated in the right panel. Uniform manifold approximation and projection (UMAP) plots displaying identified cell subsets in primary GBM (part **b**), recurrent GBM (part **c**), tumour-free SVZ (part **d**), and tumour-free cortex (part **e**) samples from patients with GBM based on snRNA-seq. Marker genes for each cluster were identified using the Wilcoxon rank-sum test (Seurat FindAllMarkers). Boxplot showing the proportions of each cluster across sample types (primary GBM, recurrent GBM, tumour-free SVZ and tumour-free cortex) (part **f**). UMAP plots displaying identified cell subsets in paired GBM (part **g**) and tumour-infiltrated SVZ (part **h**) samples from patients with GBM based on snRNA-seq. Parts **g** and h represent a matched tumour–SVZ pair from the same patient. AC, astrocyte; MES, mesenchymal; NSC, neural stem cell; OPC, oligodendrocyte precursor cell.

We investigated cell populations in the SVZ from patients with GBM to determine whether any of these populations shared genomic and transcriptional features with mGBM-OCs identified in the mouse SVZ-mutation model. A total of 140 GBM tumour tissues, 21 tumour-free cortices and 42 pathologically confirmed tumour-free SVZ tissues were obtained from supratotal resections of 94 patients with GBM and subjected to whole-transcriptome sequencing (bulk RNA-seq), together with eight SVZ tissues from patients with non-glial tumours (control SVZ) (Fig. 3a and Supplementary Table 3). Gene set enrichment analysis revealed significantly increased activity in pathways related to neuronal communication (corrected *P* <5 × 10^–26^) (Supplementary Fig. 6a) in the tumour-free SVZ of patients with GBM compared with control SVZ. Furthermore, the tumour-free SVZ of GBM displayed enhanced neuronal-like properties and enrichment of migration-associated pathways relative to GBM tumours (corrected *P* <5 × 10^–9^) (Supplementary Fig. 6b), in line with the transcriptional programmes observed in mGBM-OCs. CNA analysis based on bulk RNA-seq identified low-frequency gains of chromosome 7 and losses of chromosome 10 in the tumour-free SVZ of patients with GBM (6.3% for 7p gain; 6.3% for 10p loss; 3.1% for 10q loss) compared with the tumour-free cortex, and these alterations were more prevalent in GBM tumours (35% for 7p gain; 20% for 7q gain; 54% for 10p loss; 44% for 10q loss). Such CNAs were not observed in the control SVZ (Supplementary Fig. 6c).

To further resolve the cellular states, snRNA-seq was performed on paired samples comprising 10 tumour-free SVZ samples, 2 tumour-infiltrated SVZ samples with matched GBM tumours, a total of 15 GBM tumours (11 primary and 4 recurrent), and 4 tumour-free cortices, all derived from 10 patients with GBM, together with 2 control cortices and 2 control SVZ from 3 patients with non-glial tumours (Fig. 3a, Supplementary Fig. 7 and Supplementary Table 4). Control (non-glial tumours) SVZ and cortex samples were obtained through extended resections, following principles of microscopic total resection, as previously described^46,47^. Uniform manifold approximation and projection analysis defined six clusters: NSC-like (SOX5, SOX6), AC-like (SLC2A2, GPC5), MES-like (RUNX1, RUNX2), OPC-like (PDGFRA, TNR), cycling cells (KNL1, TOP2A, KIF15) and angiogenic (VEGFA, AKAP12) (Fig. 3b, c, Supplementary Fig. 8 and Supplementary Table 5). Each cluster was consistently mapped to the corresponding cell types (AC-like, OPC-like, NPC-like and MES-like) that were identified in a previous GBM cell population classification system (metamodule framework)^32^ (Supplementary Fig. 9a, b), except for the NSC-like cluster, which spanned all four cell types and was suggestive of progenitor-like features (Supplementary Fig. 9c–f).

EGFR and PTPRZ1, established markers associated with proliferation and stemness^48^, were enriched in NSC-like and AC-like clusters, whereas mature astrocyte and oligodendrocyte signatures were markedly depleted (Supplementary Fig. 8a). These EGFR-expressing and PTPRZ1-expressing states were detected in neuropathology-confirmed tumour-free SVZ and cortex of GBM but not in control SVZ or cortex (Fig. 3d, e and Supplementary Fig. 8b, c). In contrast, oligodendrocytes and neurons dominated control SVZ and cortex (Supplementary Fig. 8b–e and Supplementary Table 6) but constituted a smaller fraction in GBM tissues (Fig. 3f). Cluster profiles of tumour-free SVZ from GBM were further distinct from tumour-infiltrated SVZ (*n* = 2), which showed strong similarities to the tumour profiles (Fig. 3g, h), supporting the interpretation that these tumour-free SVZ samples were unlikely to be contaminated by occult tumour cells and validating their classification as non-tumour-infiltrated SVZ. To further assess intra-patient consistency, patient-wise analyses comparing tumour-free SVZ and matched tumour samples were performed as were comparisons between tumour-infiltrated SVZ and matched tumours where available (Supplementary Fig. 10a, b). Across individual patients, AC-like, NSC-like and OPC-like populations were enriched in tumours but only sparsely detected in tumour-free SVZ (Supplementary Fig. 10a). In contrast, tumour-infiltrated SVZ samples closely resembled matched tumours (Supplementary Fig. 10b), a pattern consistently observed in the Harmony-integrated analysis.

### Presence of 7-gain or 10-loss harbouring cells in the SVZ of human GBM

**Fig. 4:**
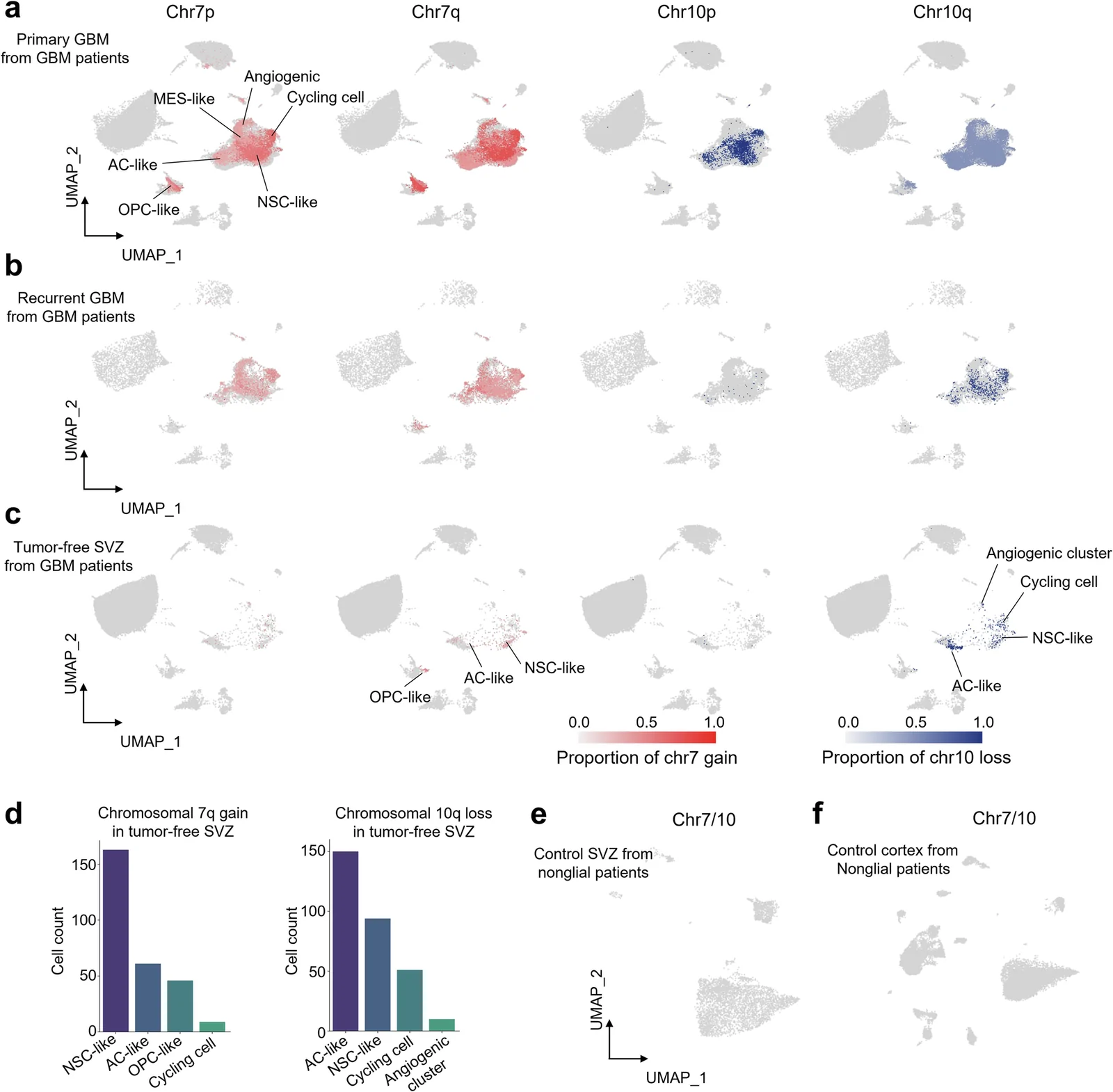
Identification of cell populations harbouring chromosome 7 gain and/or chromosome 10 loss in human GBM. Uniform manifold approximation and projection (UMAP) plots showing chromosome 7p and/or 7q arm gains and 10p and/or 10q arm losses in primary glioblastoma (GBM) (part **a**), recurrent GBM (part **b**) and tumour-free subventricular zone (SVZ) (part **c**) samples from patients with GBM. Colours indicate inferCNV-inferred, scaled copy-number signals for chromosome 7p/7q gains and chromosome 10p/10q losses. Signals were visualized using a threshold of 0.5, and chromosome-arm events were called only when five or more cells per sample met the criterion (see Methods). Bar plot showing the distribution of cell counts with chromosome 7q gain (left) or 10q loss (right) across different clusters in tumour-free SVZ samples (part **d**). Counts represent cells exceeding the same inferCNV threshold used in parts **a**–c. UMAP plots displaying chromosome 7 gain and chromosome 10 loss in control SVZ (part **e**) and control cortex (part **f**) from patients with non-glial tumours, processed identically as negative controls. AC, astrocyte; MES, mesenchymal; NSC, neural stem cell; OPC, oligodendrocyte precursor cell.

Because previous evolutionary studies have suggested that CNAs involving chromosome 7 gain and/or chromosome 10 loss represent early, clonally dominant events in GBM^11,12^, subsequent analysis assessed whether cells harbouring these CNAs could be detected in the neuropathology-confirmed tumour-free SVZ. CNA analysis of snRNA-seq data, using oligodendrocytes and microglia as controls, showed RNA-inferred signals consistent with canonical chromosome 7 gain and chromosome 10 loss in both primary and recurrent GBM across all six malignant clusters (AC-like, NSC-like, OPC-like, MES-like, cycling and angiogenic) (Fig. 4a, b, Supplementary Fig. 11a and Supplementary Table 7). Similar RNA-inferred CNA signals were observed in the tumour-free SVZ of GBM (6/10 cases, 60%), in a minor subset of cells restricted to AC-like, NSC-like and OPC-like clusters (Fig. 4c, d), but were absent in control SVZ and cortex from patients with non-glial tumours (Fig. 4e, f and Supplementary Table 8).

In matched SVZ–tumour pairs, CNA profiles of tumour-free SVZ differed from those of tumour-infiltrated SVZ, which mirrored GBM tumours in both chromosome 7/10 alterations and cluster composition (Supplementary Fig. 11b, c and Fig. 3g, h). Given that NSCs generate glial progenitor cells, OPCs and astrocyte progenitor cells, as well as NPCs^49^, these findings implicate the NSC-like population as a plausible origin-like candidate state in GBM.

Chromosomal alterations in tumour-free cortex samples from patients with GBM (*n* = 3) whose primary tumours exhibited chromosome 7 gain and chromosome 10 loss were then examined. In two cases, a minor subset of AC-like and NSC-like cells in the tumour-free cortex harboured chromosome 7 gain and chromosome 10 loss (Supplementary Fig. 12a, b). These alterations differed from patterns observed in tumour-infiltrated SVZ (Supplementary Fig. 11b, c), indicating that they were unlikely to be solely attributable to tumour infiltration. Notably, no clear chromosome 7 gain and/or chromosome 10 loss was detected in the paired tumour-free SVZ of these patients (Supplementary Fig. 12a, b). In contrast, one patient exhibited no detectable CNAs in either the tumour-free SVZ or cortex (Supplementary Fig. 12c). Given the limited cell number and coverage of tumour-free SVZ samples (*n* = 3) and the possibility of spatial heterogeneity, the presence of CNAs that were not captured by primary snRNA-seq cannot be excluded.

**Fig. 5:**
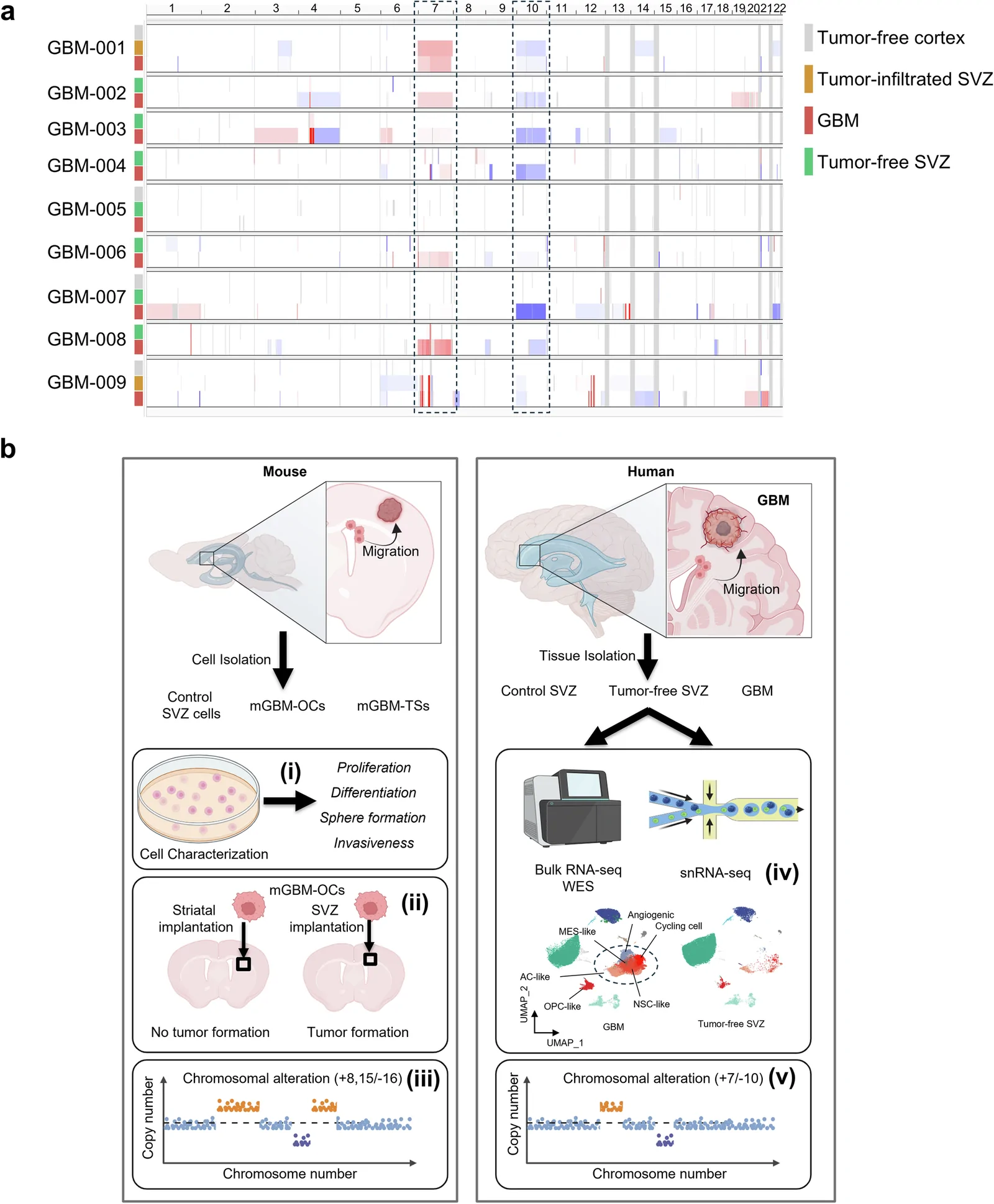
Copy-number alterations assessed by WES in patients with GBM. **a**, Copy-number alterations were inferred from high-depth whole-exome sequencing (WES) data from nine patients with glioblastoma (GBM), including seven matched tumour-free subventricular zone (SVZ)–GBM pairs (GBM-002–GBM-008) and two matched tumour-infiltrated SVZ–GBM pairs (GBM-001 and GBM-009). Tumour-free cortex samples were also included from four patients (where available). For each patient, available sample types are indicated by the coloured bars on the left (tumour-free cortex, grey; tumour-free SVZ, green; tumour-infiltrated SVZ, orange; GBM, red). The tracks show genome-wide copy-number profiles across autosomes (chromosomes 1–22), with red indicating gains and blue indicating losses (log2 copy ratio; see Methods). Dashed boxes highlight chromosomes 7 and 10. **b**, Graphical summary of this study. (1) Functional characterization of control SVZ cells, mouse glioblastoma origin-like cells (mGBM-OCs) and mouse glioblastoma tumourspheres (mGBM-TSs) in a mouse SVZ somatic mutation model. (2) Context-dependent tumorigenic potential associated with the SVZ microenvironment assessed by striatal versus SVZ implantation. (3) Accumulation of chromosomal alterations during progression from mGBM-OCs to mGBM-TSs (for example, +8, +15, −16). (4) Multi-region profiling of human tumour-free SVZ, GBM and cortex by bulk RNA sequencing (RNA-seq)/WES and single-nucleus RNA sequencing (snRNA-seq). (5) Identification of GBM-aligned SVZ stem or progenitor-like states harbouring early aneuploidy signals, including chromosome 7 gain and/or chromosome 10 loss in tumour-free SVZ.

We further assessed the presence of CNA-harbouring cell populations in the tumour-free SVZ at the DNA level. High-depth WES (average depth of coverage = 351×) of seven matched pairs of tumour-free SVZ and GBM tumours and of two matched pairs of tumour-infiltrated SVZ and GBM tumours was conducted (Supplementary Table 9). One SVZ sample (GBM-009) was termed tumour-free by neuropathology but showed CNA patterns consistent with tumour infiltration; therefore, it was analysed as tumour-infiltrated SVZ. While the majority of other CNAs observed in the tumour tissues were absent, focal amplification of chromosome 7 was observed in three cases of tumour-free SVZ (GBM-004, GBM-006 and GBM-008) (Fig. 5a), consistent with the analysis of bulk RNA-seq (Supplementary Fig. 6c). In the patient with matched snRNA-seq data (GBM-008; GN3 in the snRNA-seq), focal chromosome 7 gain in the SVZ was consistently observed across both analyses (Supplementary Fig. 13a and Supplementary Table 7). In contrast, tumour-infiltrated SVZ closely mirrored the CNA patterns of tumour tissues (Fig. 5a). This result suggests that some RNA-inferred chromosomal alterations were also detectable at the DNA level in matched tissue samples, providing tissue-level support for the snRNA-seq-based CNA inference (Fig. 4a–c).

Finally, the frequency of shared mutations between tumour-free SVZ and GBM samples from patients with GBM was analysed. Among the seven tumour-free SVZ samples, four cases harboured a low proportion of shared mutations with their paired GBM samples (Supplementary Fig. 13b), consistent with a previous report^15^. Conversely, deep sequencing of the two tumour-infiltrated SVZ samples revealed high tumour purity with substantial shared mutations, as expected (Supplementary Fig. 13c). In GBM-002, no shared mutations were detected between the tumour-free SVZ and GBM samples, yet the tumour-free SVZ sample exhibited chromosome 7 gain and chromosome 10 loss in the snRNA-seq data (Supplementary Fig. 13d). Despite the risk of sampling heterogeneity, this is compatible with the possibility that chromosomal alterations may precede somatic mutations during tumorigenesis.

Overall, this study provides evidence that chromosomal alterations are detectable at an early disease-associated stage in the SVZ and become more pronounced as the disease progresses to GBM in both mice and humans. Furthermore, it found that NSC-like, AC-like and OPC-like cells were the main cell states showing RNA-inferred chromosome 7 gain and/or chromosome 10 loss signals in both human SVZ and GBM, highlighting these populations as putative human GBM-OC-like populations (Fig. 5b).

## Discussion

Building on previous evidence that a subset of human GBMs can arise from SVZ cells harbouring low-level driver mutations^15,17^, several subsequent studies sought to leverage the SVZ concept to improve patient prognosis, although with limited success^10,50–52^. The frequent distant recurrences^10^, which develop away from the primary tumour site and often result in worse clinical outcomes, highlight the need to understand the SVZ as a potential origin-associated niche and to define the specific characteristics of GBM-associated SVZ populations. In the mouse SVZ somatic mutation model, time-series samples were collected, and it was found that SVZ-derived cells exhibit NSC-like features with distinct properties, including invasive potential and context-dependent tumorigenic potential associated with the implantation environment (Fig. 1e, f). In human patients, SVZ-resident cell populations exhibited GBM-associated transcriptional programmes and early chromosomal alterations. Taken together, these cross-species analyses support three related observations. In the mouse model, CNAs are already detectable before overt tumour formation in mGBM-OCs. In human patients, chromosome 7 gains and/or chromosome 10 losses are observed in the SVZ, and SVZ-resident AC-like, NSC-like and OPC-like populations exhibit GBM-associated transcriptional programmes and early chromosomal alterations, consistent with origin-like features. As NSCs can differentiate into OPCs and astrocyte progenitor cells^49^, the NSC-like population identified in the present study may constitute a plausible origin-like candidate state in GBM, and NSC-like, AC-like and OPC-like cells together may represent a continuum of related origin-like states that share early GBM-associated CNA patterns.

This study has several limitations. First, both mouse and human samples were used, as these two species have different chromosome numbers and gene arrangements, making it challenging to directly translate the findings from mouse models to human GBM. However, the fact that similar CNA events were observed in both species suggests that these results are directionally consistent across species (Fig. 2c, d). Second, the difficulty in obtaining paired SVZ and GBM samples from human patients limited the number of samples available for snRNA-seq. Moreover, although mGBM-OCs from the SVZ of the somatic mutation model were successfully cultured, attempts to culture human ventricular spheres from the tumour-free SVZ of patients with GBM were hampered by difficulties in obtaining and maintaining these cells. Consequently, it was not possible to genetically manipulate or thoroughly examine the cellular characteristics of human ventricular spheres and, instead, their properties were investigated using RNA-seq and DNA sequencing (Figs. 3–5). Third, while RNA-inferred CNA analysis is effective for detecting large-scale chromosomal alterations, it has limited sensitivity for focal copy-number events; moreover, because clonal evolution from SVZ populations to GBM was not directly demonstrated, definitive lineage relationships between SVZ-resident populations and fully developed tumours cannot be established. In addition, RNA-derived copy-number inference from single-nucleus transcriptomic data is subject to limitations, including transcriptional noise and dropout, particularly in low-abundance cell populations. As a result, the detection of CNAs in rare SVZ-resident cells should be interpreted as indicative rather than definitive, especially for ultra-low-frequency clones. Although concordant alterations were observed in bulk WES data, such tissue-level evidence does not by itself establish the presence of CNAs within specific rare SVZ-resident cell populations. Future studies incorporating orthogonal approaches, such as spatially resolved DNA-based methods or ultra-deep targeted sequencing, will be required to more definitively validate the presence of these CNAs within rare SVZ-resident cell populations. Fourth, genome editing using CRISPR–Cas9 and electroporation has been associated with DNA damage and chromosomal instability in certain contexts^53,54^. Although a relatively low CNA burden was observed in early-stage mGBM-OCs, suggesting that major chromosomal alterations become more prominent during tumour progression, a potential contribution of genome-editing-associated genomic instability cannot be fully excluded. Therefore, the progressive CNA patterns observed from mGBM-OCs to mGBM-TSs should be interpreted as being consistent with, rather than definitive proof of, tumour progression, while recognizing this technical possibility as a limitation. Fifth, while mGBM-OCs failed to initiate tumours following striatal implantation, the mechanistic basis for this difference remains unclear, as current data do not distinguish between potential contributions from cell survival, proliferation, apoptosis, engraftment efficiency, spatial distribution or microenvironmental support. Future studies incorporating early post-implantation analyses, including short-term survival, proliferation or apoptosis staining, spatial distribution mapping, and quantitative engraftment assessment, will be required to further clarify these possibilities. Importantly, the risk of occult tumour contamination was mitigated by (1) neuropathology confirmation of tumour-free SVZ and (2) explicitly separating tumour-infiltrated SVZ samples based on concordant cluster and CNA patterns. Nevertheless, SVZ regional assignment in the experimental system was primarily inferred from targeted microdissection combined with in vitro NSC-marker expression and functional assays, rather than direct spatially resolved validation. Although these approaches support the SVZ assignment of the isolated cells, future studies using spatial transcriptomics will be required to definitively confirm regional assignment and fully exclude potential contributions from adjacent regions.

In conclusion, in the mouse model, mGBM-OCs with stem-like or progenitor-like features and context-dependent tumorigenic potential were identified, together with progressive accumulation of CNAs during tumour progression. In human patients, rare AC-like, NSC-like and OPC-like SVZ populations with GBM-associated transcriptional programmes and early chromosomal alteration signals were identified. It is proposed that SVZ-resident AC-like, NSC-like and OPC-like populations may represent GBM-OCs exhibiting early GBM-associated chromosomal alterations (Figs. 3 and 4). Importantly, while these findings are consistent with the presence of origin-like populations in the SVZ, they do not establish direct clonal lineage relationships between these cells and GBM. These findings extend previous evolutionary reconstructions^11,12^ that positioned chromosome 7 gain and/or chromosome 10 loss in tumour-free tissue as an early, clonally dominant event in GBM by localizing such alterations to specific SVZ-resident cell states. Further studies utilizing single-cell DNA sequencing and lineage-resolved phylogenomic approaches to directly capture the mutation-bearing cells in the SVZ could provide definitive evidence for the cells of origin in GBM. A deeper understanding of these SVZ-resident GBM-associated origin-like populations will facilitate the development of novel therapeutic strategies targeting origin-like populations, potentially leading to more effective treatments for GBM.

## Supplementary information

## Data Availability

RNA sequencing data from mouse models and human whole-exome sequencing data have been deposited in the Sequence Read Archive (SRA) and are publicly available under accession numbers PRJNA1281712 and PRJNA470641, respectively. Single-nucleus RNA sequencing and bulk RNA sequencing data from human samples have been deposited in the National Center for Biotechnology Information (NCBI) Gene Expression Omnibus (GEO) and are accessible through GEO Series accession numbers GSE301788 and GSE301596, respectively. Patient information and datasets used in this study are provided in Supplementary Tables 1–8. This study used custom Python scripts and open-source R packages (R version 4.3.2). The code used for the analysis has been deposited at: https://github.com/hoonbiolab/yugbmoc_paper.
