# Supplementary material for: Putative glioblastoma origin-like cells in the subventricular zone: isolation and characterization
Source: Exp Mol Med. 2026 Aug 6;58(8):2719–32. doi: 10.1038/s12276-026-01801-4 (PMC13538383; doi:10.1038/s12276-026-01801-4)
Supplement: Supplementary file 1 [file 12276_2026_1801_MOESM1_ESM.pdf]

Putative glioblastoma origin-like cells in the subventricular zone: isolation and characterization

This supplementary information includes:

1. Supplementary methods

2. Supplementary Figs. 1–13

Supplementary Methods

Mouse bulk RNA-seq analysis

From the pre-processed RNA BAM files, the number of reads per gene was counted using FeatureCounts (v2.0.3)[1]. The raw count data were then input into DESeq2 (v1.30.1)[2] with group labels for control SVZ cells, mGBM-OC, and mGBM-TS. These data were converted to normalized values using variance stabilizing transformation implemented in DESeq2. The variance stabilizing transformation values were used for subsequent transcriptomic analyses, including differentially expressed gene (DEG) analysis, principal component analysis (PCA), and gene set enrichment analysis (GSEA). DEG analysis was performed using the DESeq() function of the DESeq2 R package. PCA was conducted with the plotPCA() function of the DESeq2 R package.

Mouse cell preparation and analysis for single cell RNA-seq

Control SVZ-C1, mGBM-TS-T1, and mGBM-OCiTS-1 and -2 cells were isolated from Trp53/Pten/hEGFRvIII-mutant mouse models and cultured in complete medium consisting of DMEM/F-12 supplemented with B27 (1X; Invitrogen), 20 ng/mL bFGF, 20 ng/mL EGF, and 1% penicillin–streptomycin. Upon formation, the spheres were collected and dissociated using Accutase solution (A6954, Sigma-Aldrich) for 1 min. The resulting single-cell suspension was washed three times with 0.04% bovine serum albumin in phosphate-buffered saline. Cell viability was assessed using trypan blue staining, and only viable cells were utilized for scRNA-seq.

scRNA-seq libraries were prepared using a droplet-based platform (details below). Raw sequencing data were processed using Cell Ranger (v3.1.0; 10× Genomics) and aligned to the GRCm38 reference genome. Normalization and scaling of the scRNA-seq data were conducted using the NormalizeData and ScaleData functions in the Seurat package (v.5.1.0). We identified 3,000 highly variable genes for initial clustering using the FindVariableFeatures function. Batch correction was performed with the Harmony package (v.1.2.0). For dimensionality reduction, UMAP was applied using 30 Harmony dimensions, with n.neighbors set to 30 and min.dist set to 0.3. Cell clusters were identified using the FindNeighbors and FindClusters functions, with the resolution parameter set to 0.3. Gene set enrichment analysis was conducted using the fgsea package (v.1.28.0) to compare mGBM-TS-T1 and Control SVZ-C1, as well as

mGBM-OCiTS-1 and -2 against Control SVZ-C1, using hallmark gene sets from the msigdbr package (v.7.5.1).

Single-nucleus isolation of human samples

GN- or GC- samples were obtained from patients with GBM. CN- samples were from patients with non-glial tumors. Single nuclei were isolated from fresh-frozen tissue samples according to a previous report with minor modifications[3]. Briefly, tissue samples were pulverized with scalpels in lysis buffer [0.2% Triton X-100, 1X protease inhibitor, 1 mM dithiothreitol, 0.2 U/μL RNAsin (Promega), 2% (w/v) bovine serum albumin in phosphate-buffered saline]. Homogenized tissue samples were filtered using 40 μm strainers, after which DAPI (0.5 μg/mL) was added to stain nuclei. Free nuclei were again filtered using a 20 μm pluriStrainer Mini and analyzed using fluorescence-activated cell sorting (BD FACSAria™ system, BD). Single nuclei from the DAPI-positive population were sorted for subsequent snRNA-seq.

Library preparation for snRNA-seq

All snRNA-seq libraries were prepared using Chromium Next GEM Single-cell 3' GEM, Library & Gel Bead Kit, V3.1 (10× Genomics) according to the manufacturer's instructions. The initial step consisted of creating an emulsion in which individual nuclei were isolated into droplets together with gel beads coated with unique primers bearing 10× cell barcodes, unique molecular identifiers, and poly(dT) sequences. Reverse transcription reactions were performed to generate barcoded full-length cDNA, followed by the disruption of emulsions using the recovery agent and cDNA clean-up using DynaBeads MyOne Silane Beads (10× Genomics). Bulk cDNA was amplified using a SimpliAmp Thermal Cycler (98 °C for 3 min; cycled 11–13 times (according to targeted cell recovery): 98 °C for 15 s, 67 °C for 20 s, and 72 °C for 1 min; 72 °C for 1 min; held at 4 °C). The amplified cDNA product was cleaned using the SPRIselect Reagent Kit (Beckman Coulter). Indexed sequencing libraries were constructed using the reagents from the Chromium Single-cell 3 v3.1 Reagent Kit, as follows: fragmentation, end repair, and A-tailing; size selection with SPRIselect; adaptor ligation; post-ligation clean-up with SPRIselect; sample index polymerase chain reaction and clean-up with SPRIselect beads. Library quantification and quality assessment were performed on 4200Tapestation with an HSD1000 ScreenTape (Agilent Genomics). Indexed libraries were sequenced on an Illumina HiSeq series using paired-end 26×98 bp.

Human bulk RNA-seq gene enrichment analysis

First, the raw FASTQ files were analyzed with FastQC (v0.11.9)[4] to assess the overall sequencing quality and possible adapter sequence contamination. Skewer (v0.11.9)[5] was used to remove Illumina TruSeq adapter sequences (read 1: 5′-AGATCGGAAGAGCACACGTCTGAACTC-CAGTCA-3′, read 2: 5′-AGATCGGAAGAGCGTCGTGTAGGGAAAGAGTGT-3′) from the read files. Subsequently, the reads were aligned to the GRCh38 (hg38) reference genome using the STAR aligner (v2.7.10a)[6] to generate coordinate-sorted BAM files. FeatureCounts (v2.0.3)[1] was used to generate the gene expression count table, which was subsequently normalized to transcripts per million (TPM) using R (v4.3.3). Differential expression analysis was performed using the DESeq2[2] R package, and the genes that met the following threshold were considered significant: $|\log_2 \text{fold change}| > 0.5$ and Benjamini–Hochberg (BH)-adjusted P-value < 0.05. Pathway enrichment analyses for GO Biological Process were then conducted using the clusterProfiler[7] R package.

Human bulk RNA-seq copy number analysis

STAR aligner (v2.5.3)[6] was used to map RNA reads to the hg19 reference genome, and the read count file was generated. Base quality recalibration, variant calling, and filtering were performed using the GATK pipeline (v4.1.8.1), generating a VCF file. The read count and VCF files were used as input for RNAseqCNV (v0.0.4)[8] R package to estimate chromosomal arm-level CNVs.

Values of $\log_2$ fold change per chromosome arm from RNAseqCNV was visualized as a heatmap. In this process, 17 tumor-free cortex, 8 tumor-free SVZ, 32 control SVZ, and 111 GBM samples were included for RNAseqCNV. The average $\log_2$ fold change from the tumor-free cortex samples was regarded as a background effect, which was subtracted from the fold changes in the SVZ, control SVZ, and GBM samples.

References

1. Liao, Y., Smyth, G. K. & Shi, W. featureCounts: an efficient general purpose program for assigning sequence reads to genomic features. *Bioinformatics.* 30, 923–930 (2014).
2. Love, M. I., Huber, W. & Anders, S. Moderated estimation of fold change and dispersion for RNA-seq data with DESeq2. *Genome Biol.* 15, 550 (2014).
3. Baslan, T. et al. Genome-wide copy number analysis of single cells. *Nat Protoc.* 7, 1024–1041 (2012).
4. Andrews, S. FastQC: A Quality Control Tool for High Throughput Sequence Data. http://www.bioinformatics.babraham.ac.uk/projects/fastqc/ (2010).
5. Jiang, H., Lei, R., Ding, S. W. & Zhu, S. Skewer: a fast and accurate adapter trimmer for next-generation sequencing paired-end reads. *BMC Bioinformatics.* 15, 182 (2014).
6. Dobin, A. et al. STAR: ultrafast universal RNA-seq aligner. *Bioinformatics.* 29, 15–21 (2013).
7. Wu, T. et al. clusterProfiler 4.0: A universal enrichment tool for interpreting omics data. *Innovation (Camb).* 2, 100141 (2021).
8. Barinka, J. et al. RNAseqCNV: analysis of large-scale copy number variations from RNA-seq data. *Leukemia.* 36, 1492–1498 (2022).

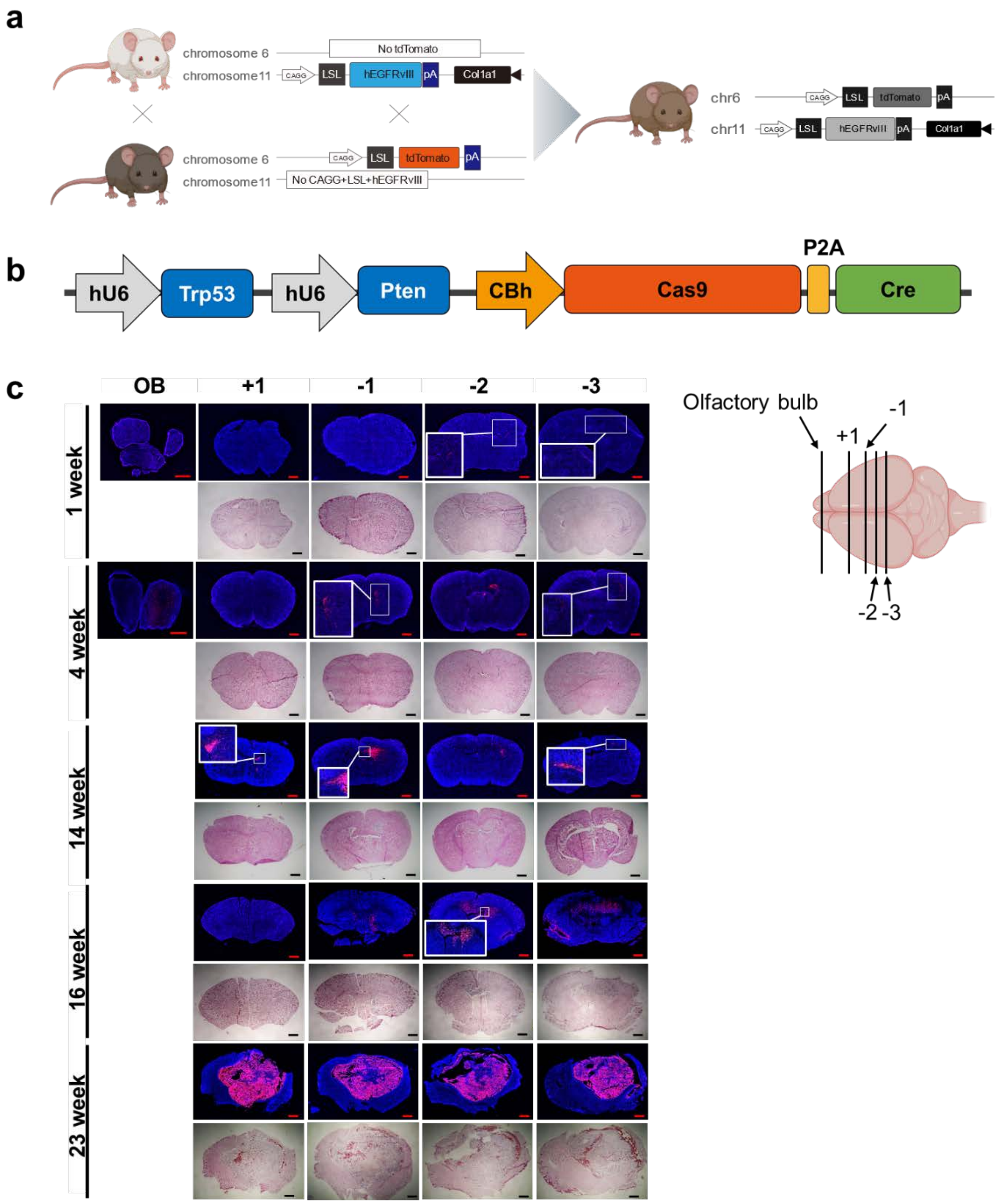

Supplementary Fig. 1 Serial observation of GBM development using the SVZ somatic mutation mouse model. a Schematic overview of the mouse model generated by breeding LSL-hEGFRvIII mice with LSL-tdTomato mice. **b** Plasmid Map used to express Cas9, Cre recombinase, and sgRNAs targeting *Trp53* and *Pten*. **c** Serial coronal sections of mouse brains illustrating GBM development after SVZ electroporation. Whole-section fluorescence images (tdTomato and DAPI counterstain) and matched H&E sections show tdTomato-positive cells

initially confined to the SVZ, followed by tumor formation over time. Time points are indicated in each panel. Scale bars: 1 mm.

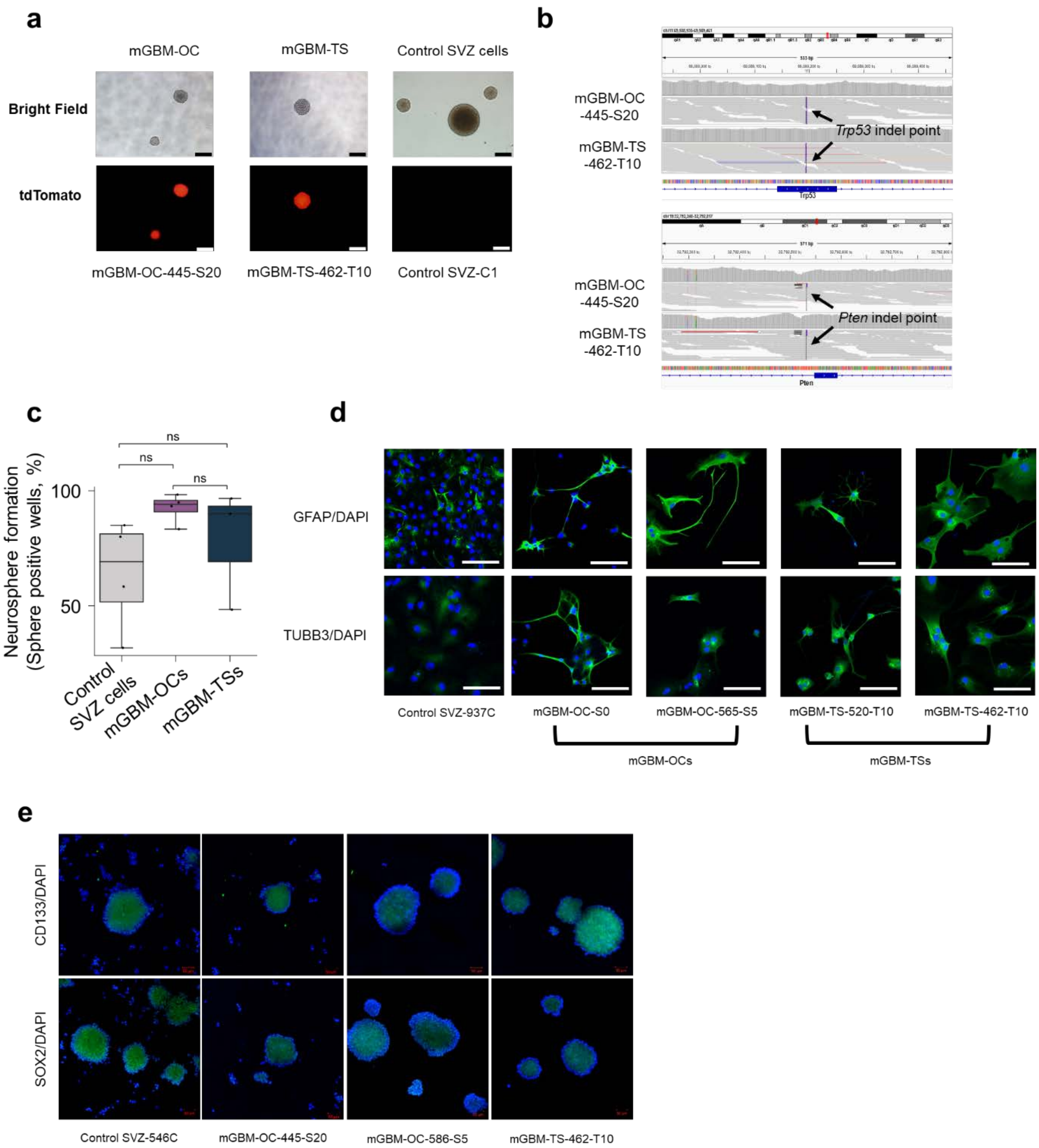

Supplementary Fig. 2 Characterization of putative mouse glioblastoma origin-like cells (mGBM-OCs) and mouse glioblastoma tumorspheres (mGBM-TSs) a Representative brightfield (top) and tdTomato fluorescence (bottom; red) images of mGBM-OCs, mGBM-TSs, and control SVZ cells (left to right). Control SVZ cells are tdTomato-negative. Scale bar: 200 μm. **b** Representative IGV (Integrative Genomics Viewer) views from WGS showing

indels in *Trp53* and *Pten* across mGBM-OC-445-S20 and mGBM-TS-462-T10. Indels were consistently detected across independent mGBM-OCs and mGBM-TSs lines (see Supplementary Table 1). **c** Comparison of neurosphere formation capacity among the three groups (control SVZ cells, mGBM-OCs, and mGBM-TSs). Boxes represent the 25th, 50th (median), and 75th percentiles, and whiskers extend to 1.5 times the interquartile range. Each dot represents one biological replicate (independent cell line). **d** Neuroglial differentiation of three groups. Representative immunofluorescence images after differentiation showing neuronal (TUBB3) and astrocytic (GFAP) markers in control SVZ cells, mGBM-OCs, and mGBM-TSs. TUBB3 and GFAP are shown in green with DAPI (blue) counterstain. GFAP, glial fibrillary acidic protein; TUBB3, tubulin beta 3 class III. Scale bar: 100 μm. **e** Immunocytochemical validation of neural stem cell/progenitor-associated marker expression. Representative immunofluorescence images showing expression of SOX2 and CD133 in control SVZ cells, mGBM-OCs, and mGBM-TSs. SOX2 and CD133 are shown in green with DAPI (blue) counterstain. SOX2, SRY-box transcription factor 2; CD133, prominin-1. Scale bar: 50 μm.

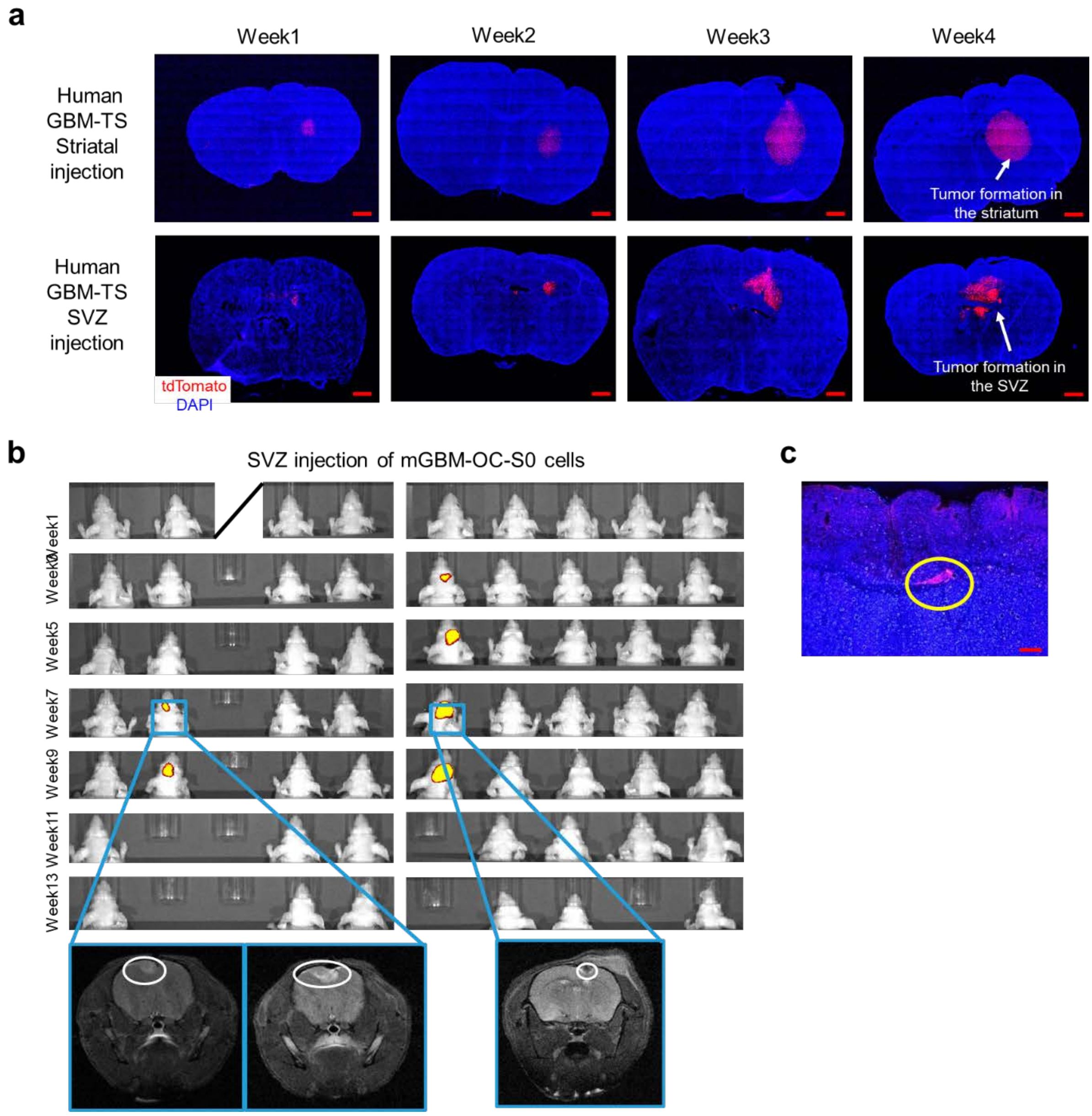

Supplementary Fig. 3 Serial observation of tumor formation. a Following implantation of human GBM-TS (TS13-64, mCherry-labeled) into the striatum or SVZ, whole-section fluorescence images (red signal with DAPI counterstain) of serial coronal brain sections show tumor formation at the respective implantation sites. Scale bar: 1 mm. **b** Representative longitudinal IVIS images and corresponding brain MRI following SVZ implantation of mGBM-OC-S0 are shown. Tumor formation was monitored by IVIS (in vivo imaging system) and confirmed by brain MRI (circled regions). **c** At 48 hour after SVZ implantation of mGBM-OCs, one mouse was harvested to verify accurate SVZ targeting by fluorescence microscopy. The yellow circle indicates tdTomato-positive cells within the SVZ area. Scale bar: 50 μm.

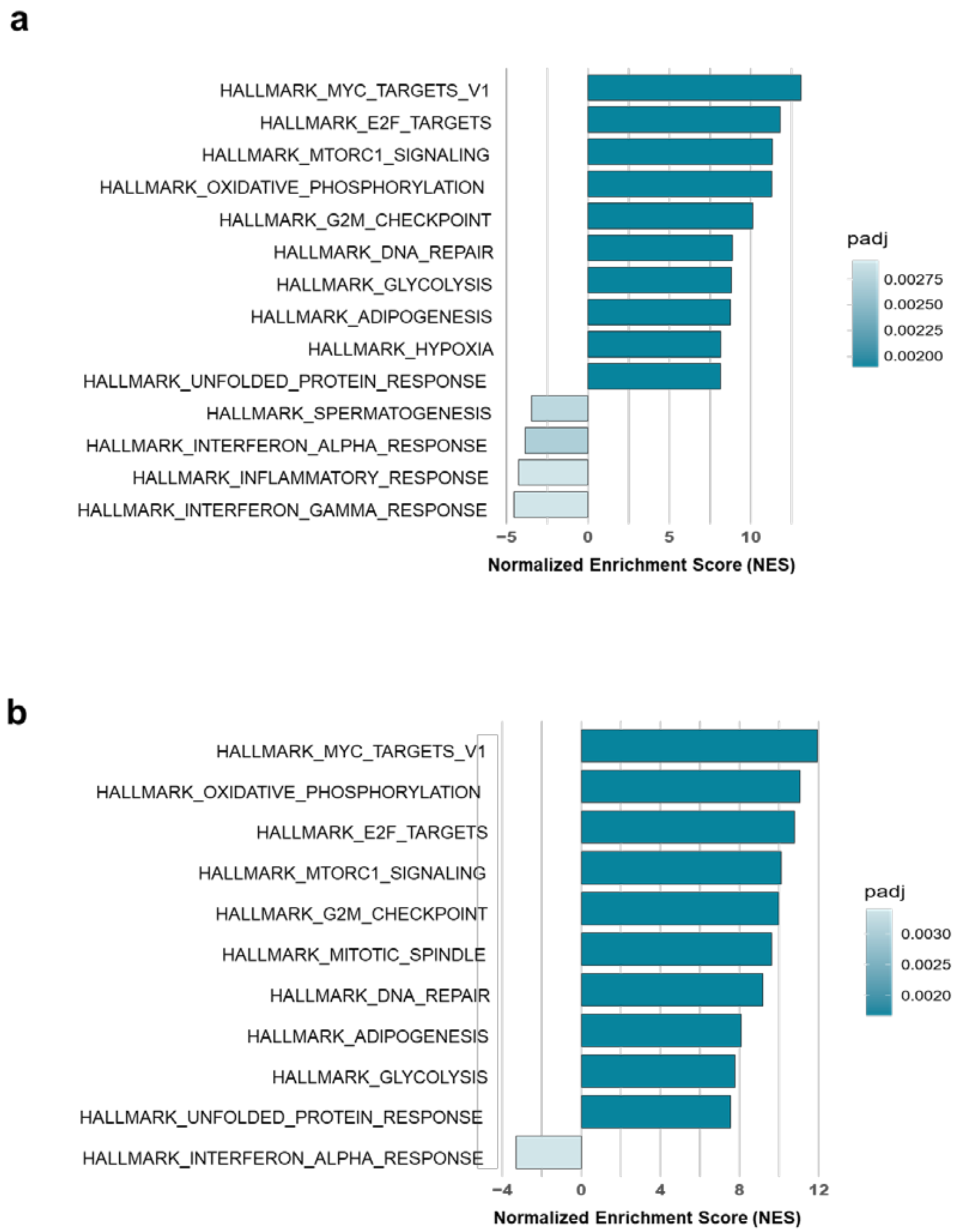

Supplementary Fig. 4 Gene set enrichment analysis (GSEA) of mouse GBM tumorspheres (mGBM-TS-T1) and tumorspheres derived from SVZ-implanted mGBM-OCs (mGBM-OCiTS-1 and -2) compared with control SVZ cells (control SVZ-C1). a, b Bar plots summarize Hallmark GSEA results for (a) mGBM-TS-T1 versus control SVZ-C1 and (b) mGBM-OCiTS-1/2 versus control SVZ-C1. Positive scores indicate gene sets enriched in the indicated tumorsphere population, whereas negative values indicate gene sets enriched in control SVZ cells. Both tumorsphere groups show enrichment of proliferation- and

metabolism-related programs.

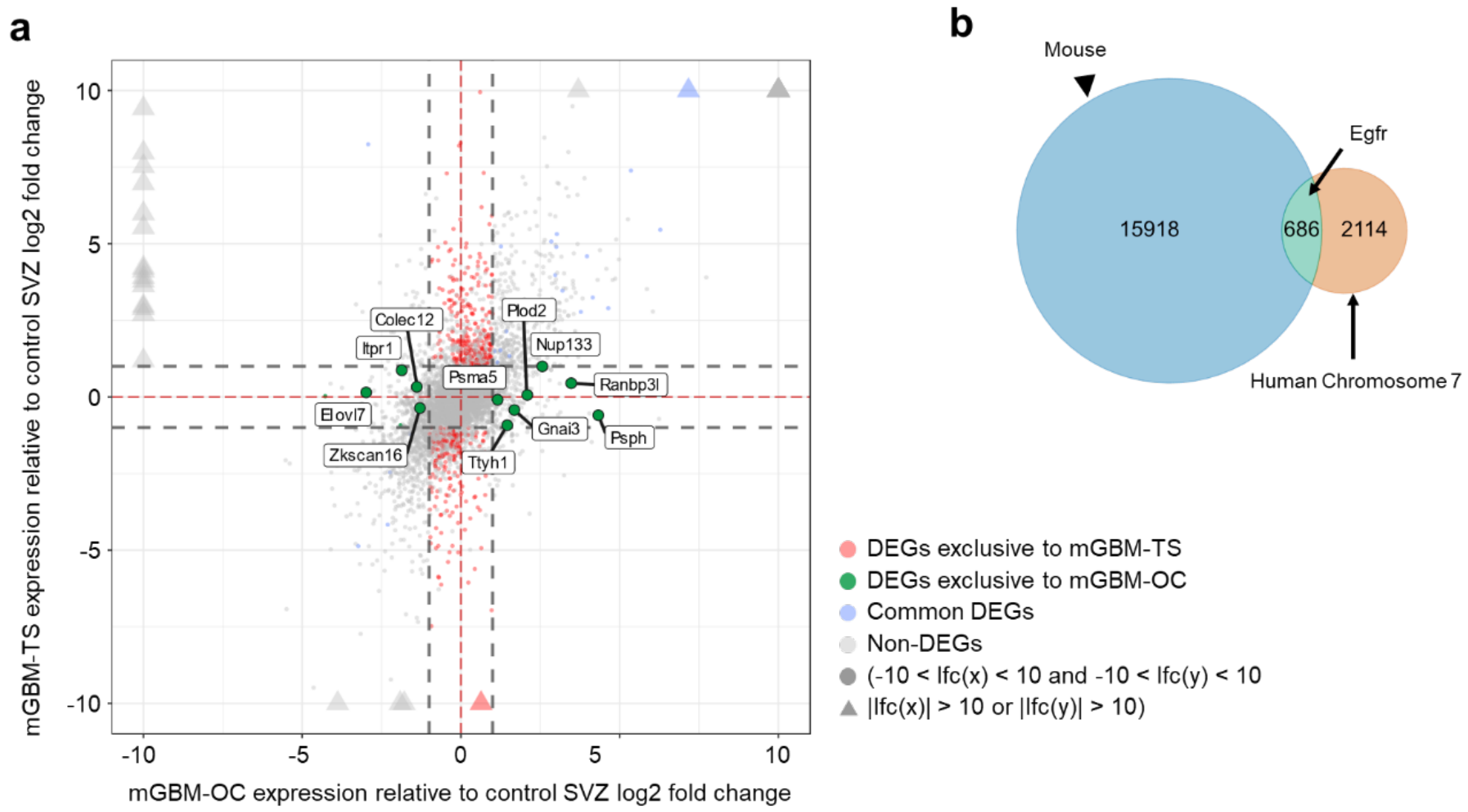

c

Mouse No.	Sex	Blood X LogRatio	Blood Y LogRatio	Blood Call	Case X Log Ratio	Case Y Log Ratio	Case Call	Sex Chromosome
mGBM-OC-445-S20	M	-0.897	+0.0182	Male	-0.961	-4.27	Female	Y-loss
mGBM-OC-565-S5	M	-0.887	+0.109	Male	-0.992	-0.651	Male	
mGBM-OC-586-S5	M	-0.897	-1.62	Male	-0.94	-4.27	Female	Y-loss
mGBM-TS-458-T20	M	-0.889	-0.297	Male	-1.02	-5.03	Female	Y-loss
mGBM-TS-462-T10	F	+0.12	-22	Female	+0.401	-21.9	Female	
mGBM-TS-468-T20	M	-0.916	-0.467	Male	-1.04	-4.56	Female	Y-loss
mGBM-TS-475-T10	M	-0.944	-1.7	Male	-0.713	-6.58	Female	Y-loss
mGBM-TS-517-T10	M	-0.937	-0.301	Male	-1.02	-1.46	Male	
mGBM-TS-520-T10	M	-0.937	-0.000223	Male	-1.14	-1.4	Male	
mGBM-TS-523-T10	F	+0.0598	-22.1	Male	+0.0321	-22.6	Male	

Supplementary Fig. 5 Differential expression and copy-number analyses of mGBM-OCs and mGBM-TSs. a Scatter plot of differential gene expression for mGBM-OCs and mGBM-TSs relative to control SVZ cells. The x-axis represents log2 fold change of mGBM-OC compared with control SVZ, and the y-axis represents log2 fold change of mGBM-TS compared with control SVZ. Each dot corresponds to an individual gene. Red, green, and blue dots indicate DEGs exclusive to mGBM-TS, exclusive to mGBM-OC, and shared between both groups, respectively; gray dots denote non-DEGs. Black dashed lines indicate log2 fold-change thresholds (|log2FC| = 1), and red dashed lines indicate log2FC = 0. DEGs were defined as |log2FC| > 1 with adjusted P-value < 0.05; genes with |log2FC| > 10 are plotted as triangles at the plot boundaries. **b** Venn diagram illustrating the number of genes with copy number

alterations in mGBM-TSs and genes located on human chromosome 7. The overlap includes Egfr. **c** Sex-chromosome status inferred from WGS-based copy-number analysis of matched blood and case samples, highlighting Y-loss events.

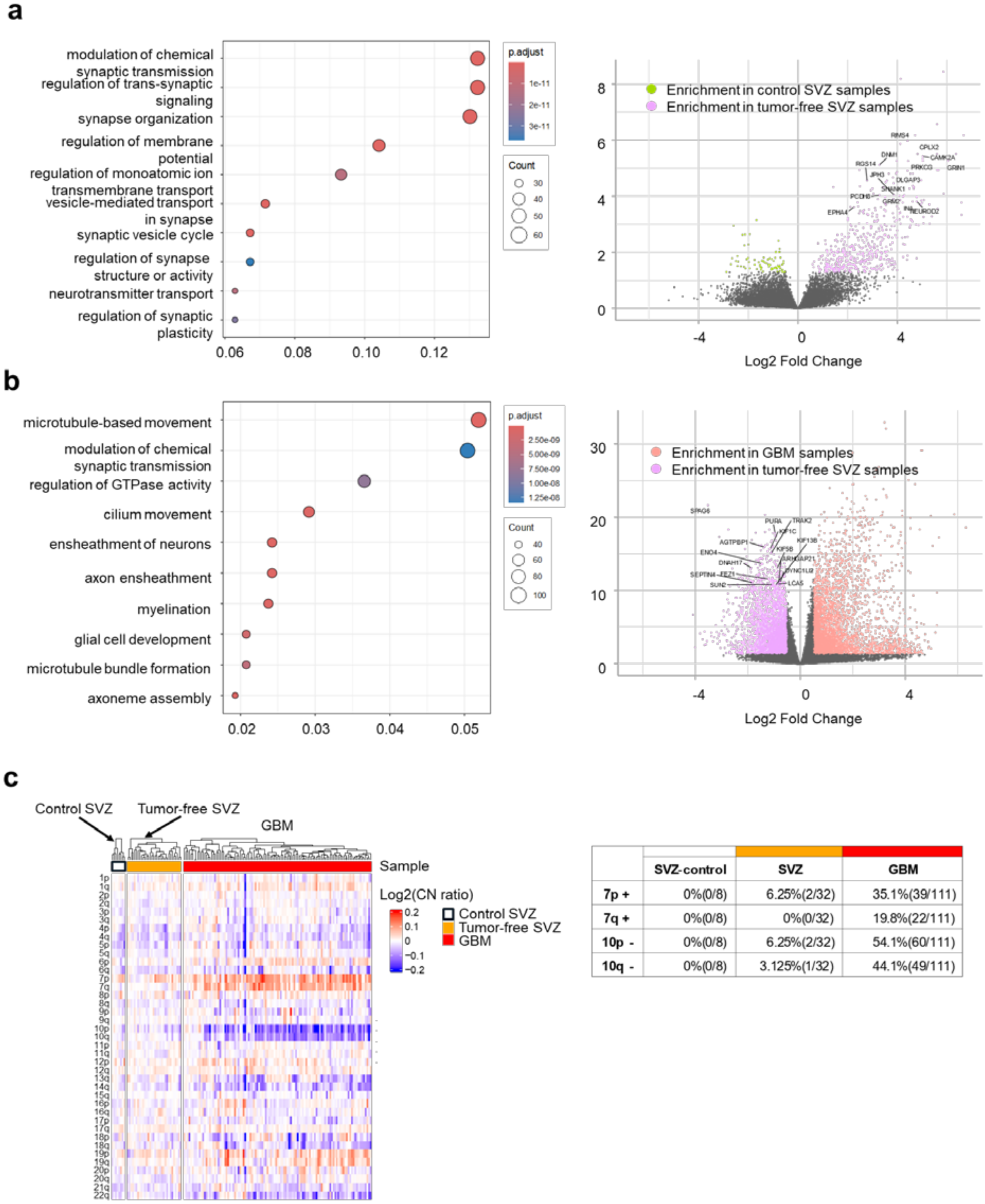

	SVZ-control	SVZ	GBM
7p +	0%(0/8)	6.25%(2/32)	35.1%(39/111)
7q +	0%(0/8)	0%(0/32)	19.8%(22/111)
10p -	0%(0/8)	6.25%(2/32)	54.1%(60/111)
10q -	0%(0/8)	3.125%(1/32)	44.1%(49/111)

Supplementary Fig. 6 Bulk RNA-seq analysis of human samples. a Gene ontology (GO) biological process enrichment (dot plot, left) and differential expression (volcano plot, right) comparing tumor-free SVZ from patients with GBM to control SVZ from patients with non-

glial tumors. Genes annotated to “modulation of chemical synaptic transmission” are highlighted in the volcano plot. **b** GO biological process enrichment (dot plot, left) and differential expression (volcano plot, right) comparing tumor-free SVZ from patients with GBM to primary GBM samples. Genes annotated to “microtubule-based movement” are highlighted in the volcano plot. **c** Arm-level copy-number profiles inferred from bulk RNA-seq data for control SVZ, tumor-free SVZ, and GBM samples (RNAseqCNV). The heatmap displays chromosome arm–level log2 fold-change values across samples, and the table summarizes the frequencies of chr7p/7q gain and chr10p/10q loss.

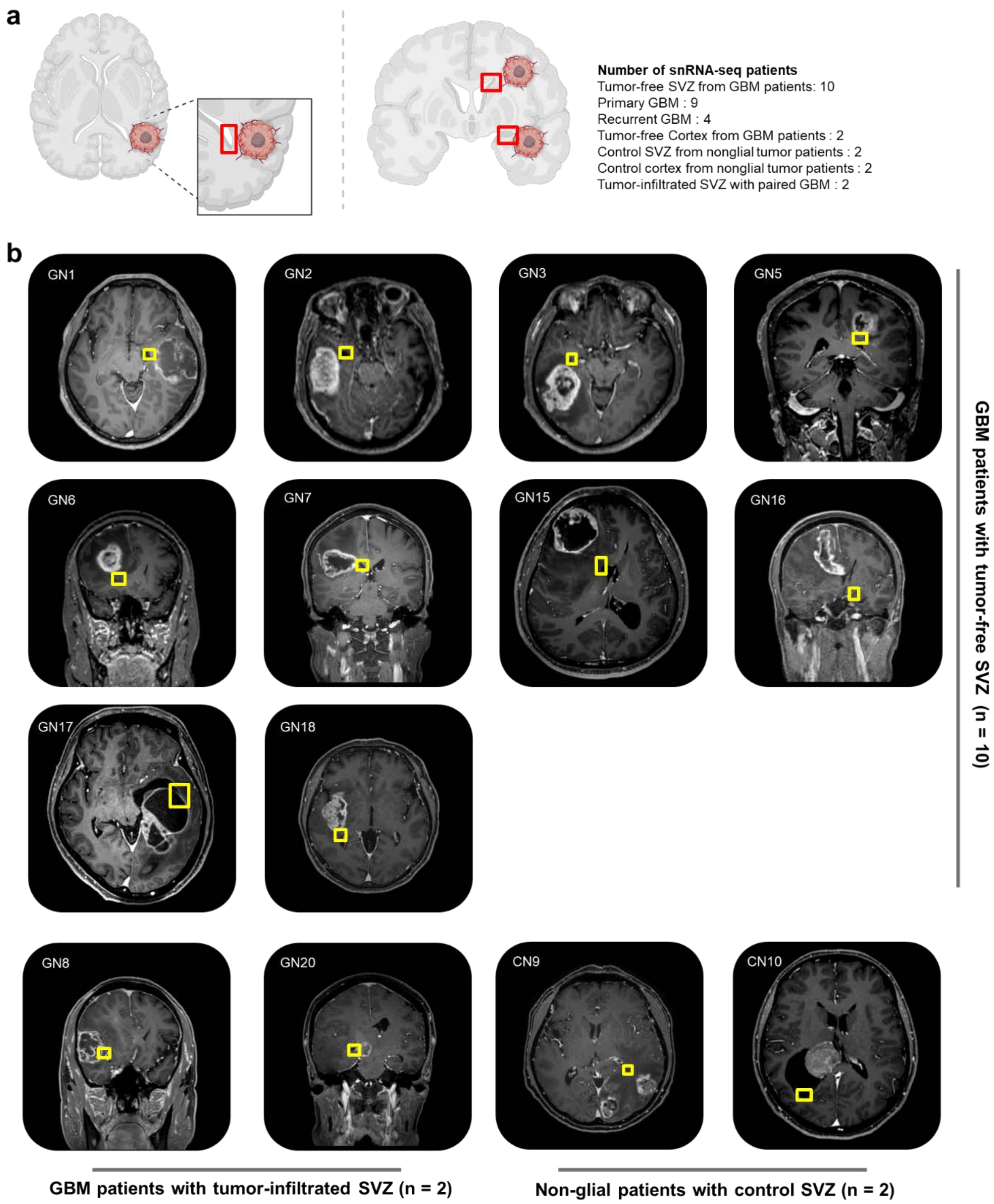

Supplementary Fig. 7 Patient MRI information for sampled patients. a Schematic illustration of the sampling strategy based on tumor location. The red squares indicate sampling sites, including the SVZ along the lateral ventricle. **b** Representative MRI images for each patient. The yellow box highlights the SVZ sampling site in each tumor case. GN denotes samples from patients with GBM, and CN denotes samples from control patients with non-glial tumors; all were analyzed by snRNA-seq (see Supplementary Table 3).

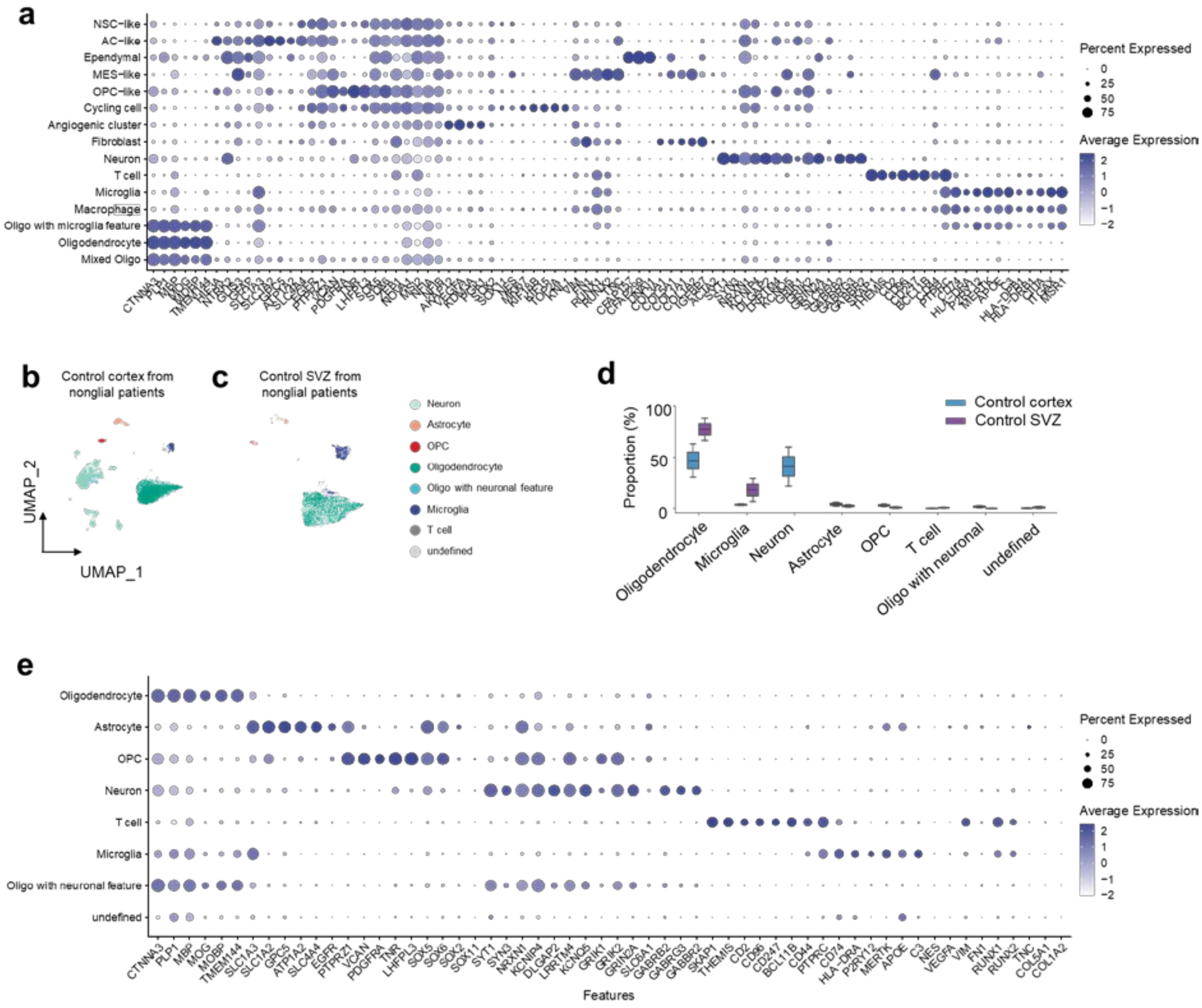

Supplementary Fig. 8 Gene expression characteristics of GBM and control (non-glial tumor) samples. a Dot plot showing selected marker genes for each cluster in the GBM patients snRNA-seq dataset (Supplementary Table 4). **b, c** UMAP plots displaying identified cell subsets in control cortex (b) and control SVZ (c) samples from patients with non-glial tumors. OPC, oligodendrocyte precursor cell. **d** Boxplot comparison showing the proportions of each cluster between control cortex and control SVZ. **e** Dot plot showing selected marker genes for each cluster in samples from patients with non-glial tumors (Supplementary Table 5).

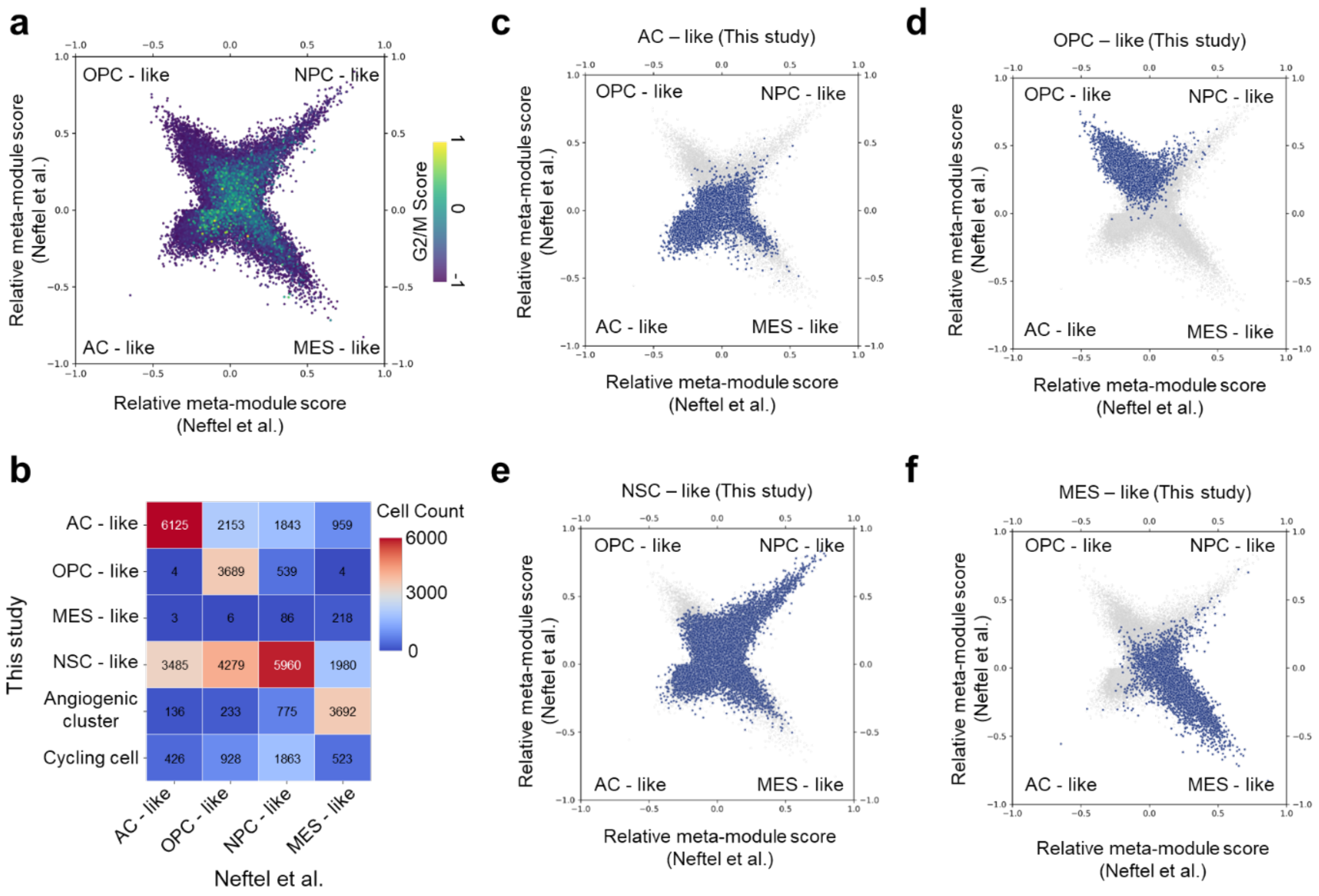

Supplementary Fig. 9 Comparison of annotated clusters with established classification. a Two-dimensional representation of cellular states. Each quadrant corresponds to a distinct cellular state, with individual cells (dots) positioned based on their relative meta-module scores. Color indicatethe density of cycling cells (Supplementary Methods). **b** Confusion matrix depicting the distribution of cell counts between annotated clusters from this study (rows) and those defined by Neftel et al (ref. 30) (columns). **c-f** Two-dimensional representation of cellular states for AC-like (**c**), OPC-like (**d**), NSC-like (**e**), and MES-like (**f**) populations identified in this study. Each quadrant represents a cellular state as defined by Neftel et al. (ref. 30), with individual cells (dots) positioned according to their meta-module scores. Cells from the indicated cluster are highlighted (blue), and all other cells are shown in light gray.

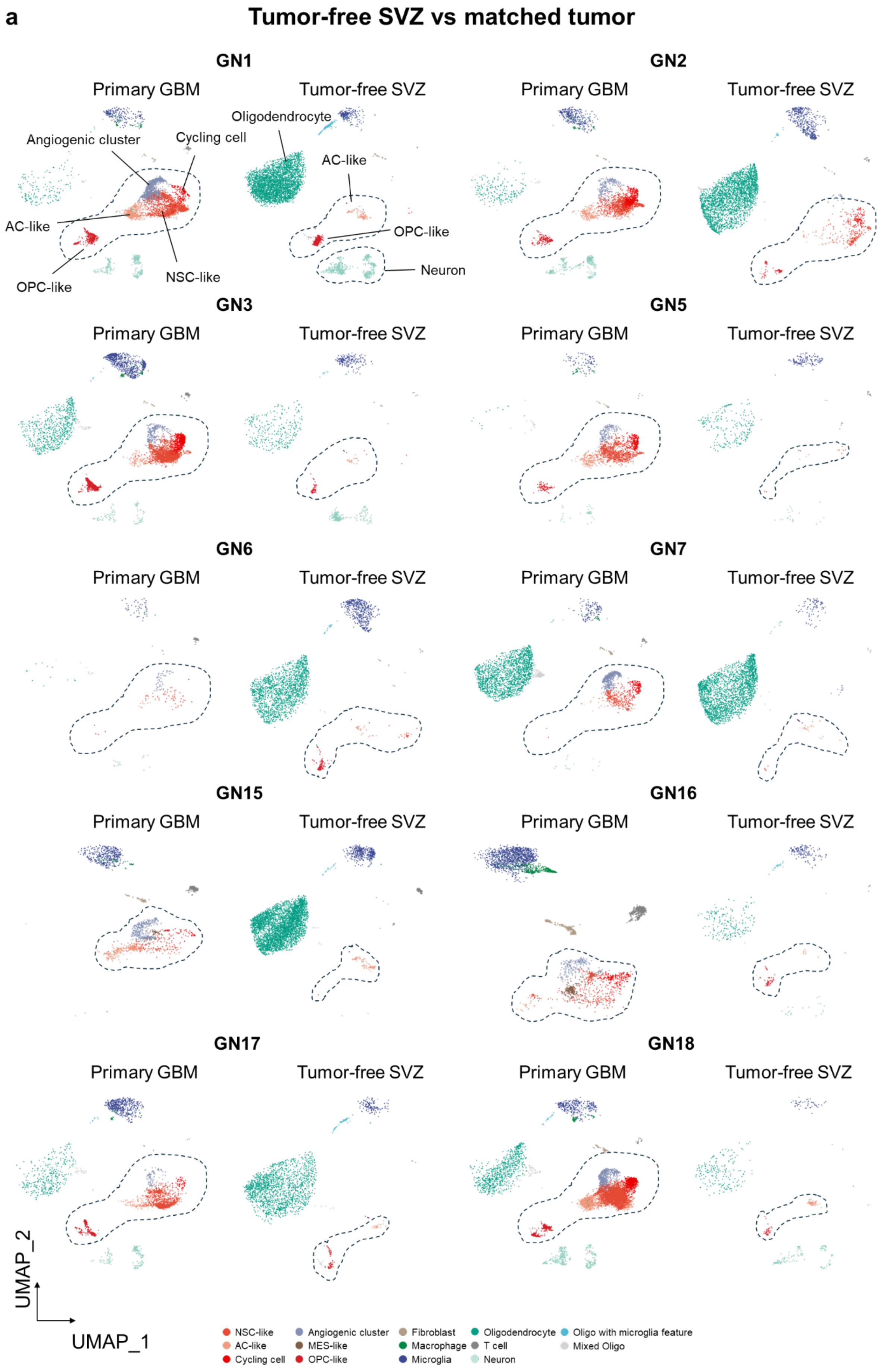

a
Tumor-free SVZ vs matched tumor
GN1
Primary GBM
Tumor-free SVZ
Angiogenic cluster
Cycling cell
AC-like
OPC-like
NSC-like
Oligodendrocyte
Neuron
GN2
GN3
GN5
GN6
GN7
GN15
GN16
GN17
GN18
UMAP_2
UMAP_1
NSC-like
AC-like
Cycling cell
Angiogenic cluster
MES-like
OPC-like
Fibroblast
Macrophage
Microglia
Oligodendrocyte
T cell
Neuron
Oligo with microglia feature
Mixed Oligo

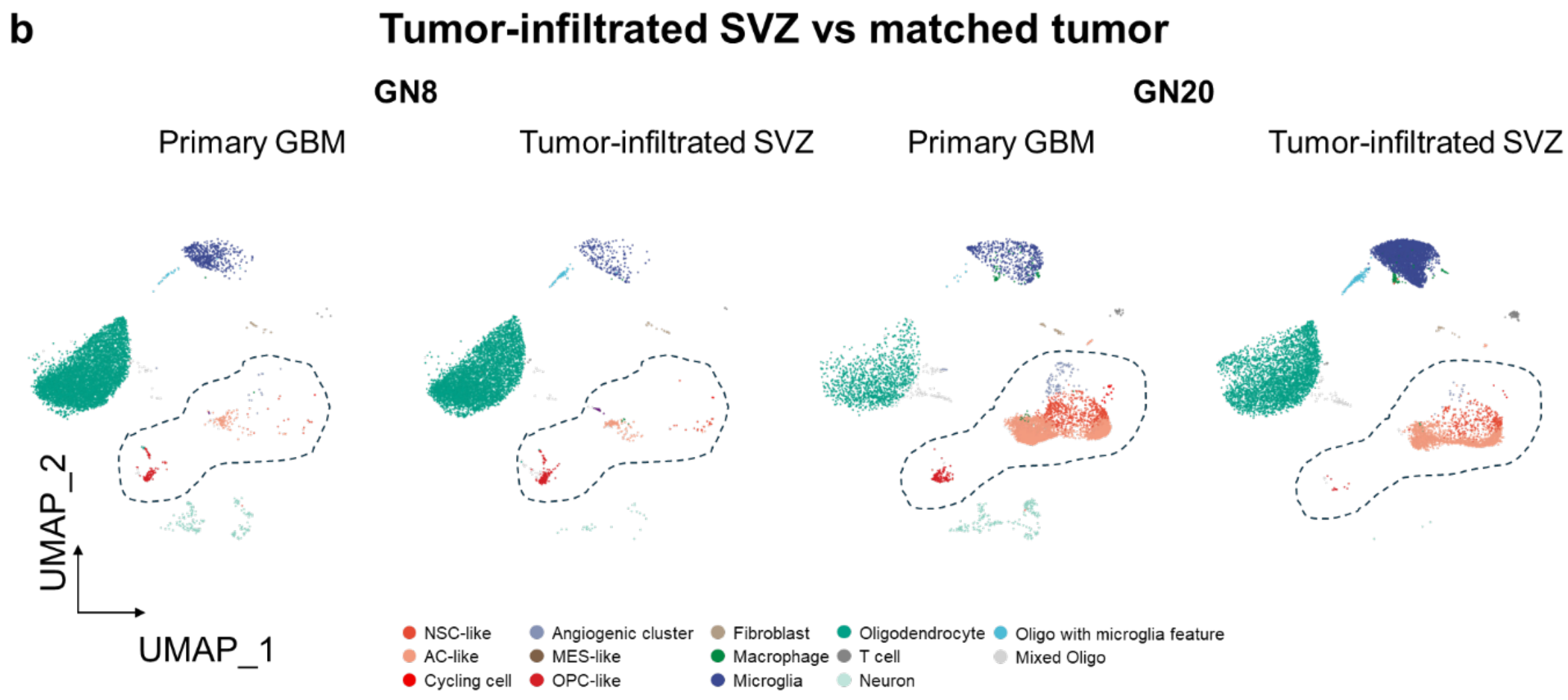

Supplementary Fig. 10 Patient-wise comparison of SVZ and matched tumor samples. a, UMAP plots showing tumor-free SVZ and matched tumor samples from individual patients with GBM. **b** UMAP plots showing tumor-infiltrated SVZ and matched tumor samples from individual patients with GBM. Samples from the same patient are shown side-by-side to facilitate direct intra-patient comparison of cluster composition in the Harmony-integrated UMAP space. Dashed outlines indicate AC-like, NSC-like, and OPC-like populations highlighted for comparison across paired samples.

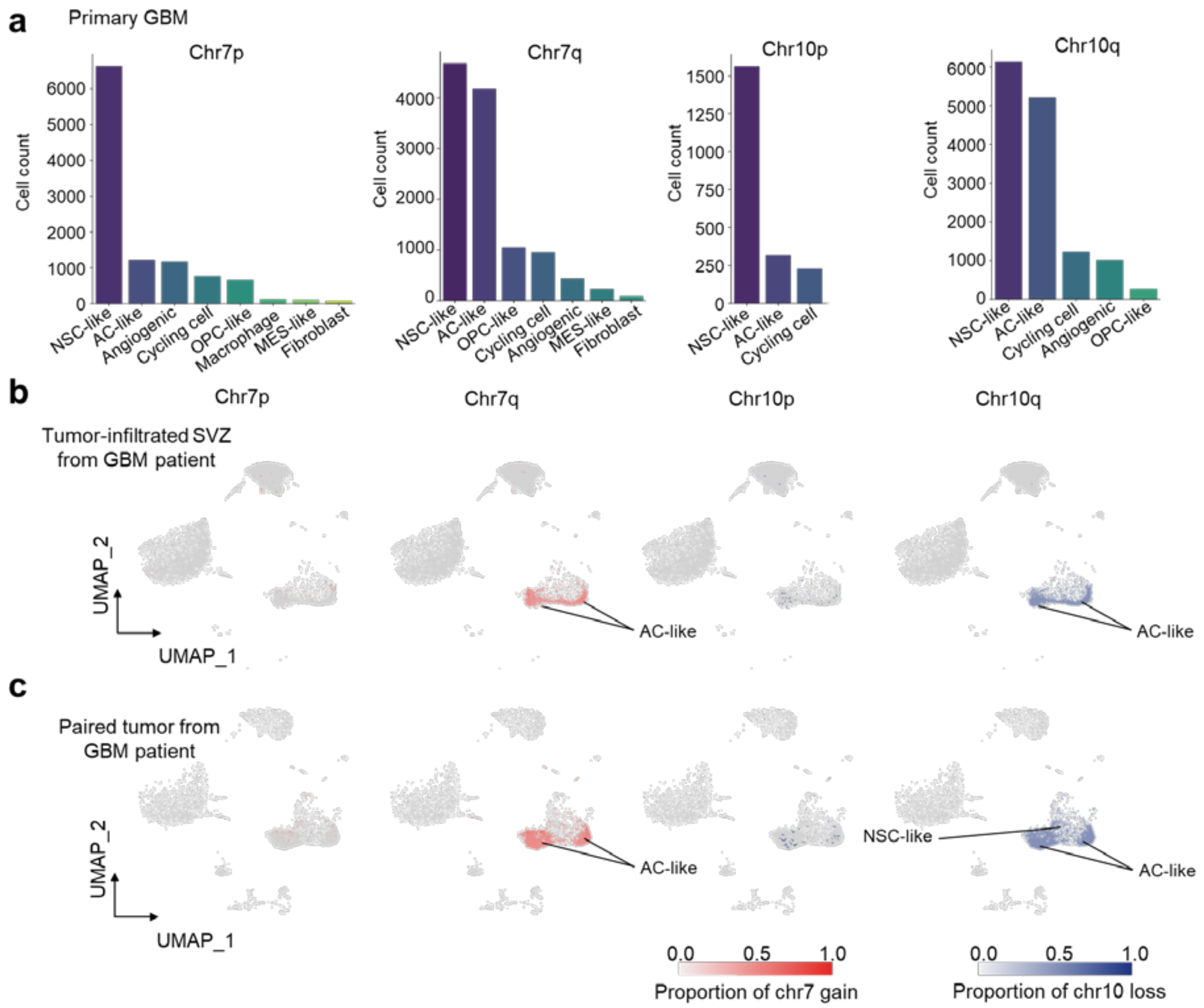

Supplementary Fig. 11 Characterization of cell populations with chromosome 7 gain and/or chromosome 10 loss in human GBM. a Bar plots (Chr7p, Chr7q, Chr10p, and Chr10q) showing the distribution of cells meeting chromosome-arm event criteria with chromosome 7p or 7q arm gain and 10p or 10q arm loss across different clusters in primary GBM. AC-like, astrocyte-like; MES-like, mesenchymal-like; OPC-like, oligodendrocyte precursor cell-like; NSC-like, neural stem cell-like. **b, c** UMAP plots showing chromosome 7p or 7q arm gain and 10p or 10q arm loss in tumor-infiltrated SVZ (**b**), and paired GBM (**c**) samples from GBM patients. Colors indicate the extent of inferCNV-inferred scaled copy-number signals for chromosome-arm gain (Chr7p/7q; red scale) or loss (Chr10p/10q; blue scale). Chromosome-arm events were called using a 0.5 threshold and only when ≥5 cells per sample met the criterion (see Methods). AC-like, astrocyte-like; NSC-like, neural stem cell-like.

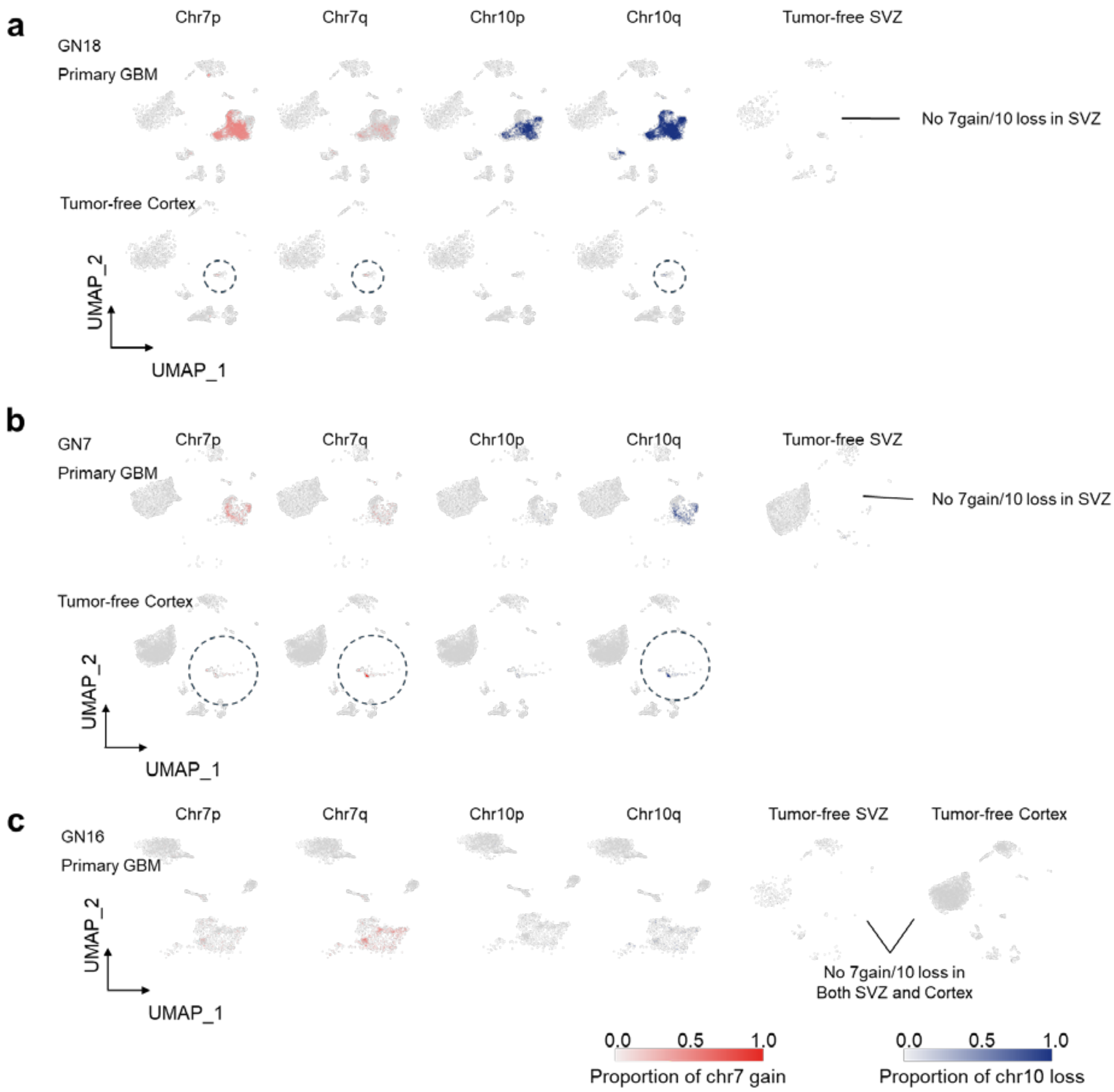

Supplementary Fig. 12 Identification of rare cell subsets with chromosome 7 gain and/or chromosome 10 loss in tumor-free cortex of patients with GBM. a-c UMAP plots overlaying inferCNV-inferred chromosome-arm copy-number signals for chromosome 7p or 7q arm gain and 10p or 10q arm loss in matched primary GBM, tumor-free cortex, and tumor-free SVZ samples from patients with GBM (**a**, GN18; **b**, GN7; **c**, GN16). Colors indicate the scaled extent of chromosome 7 gain (red) or chromosome 10 loss (blue) with gray denoting cells not meeting the event threshold. Circles highlight CNA-positive cells detected in the tumor-free cortex.

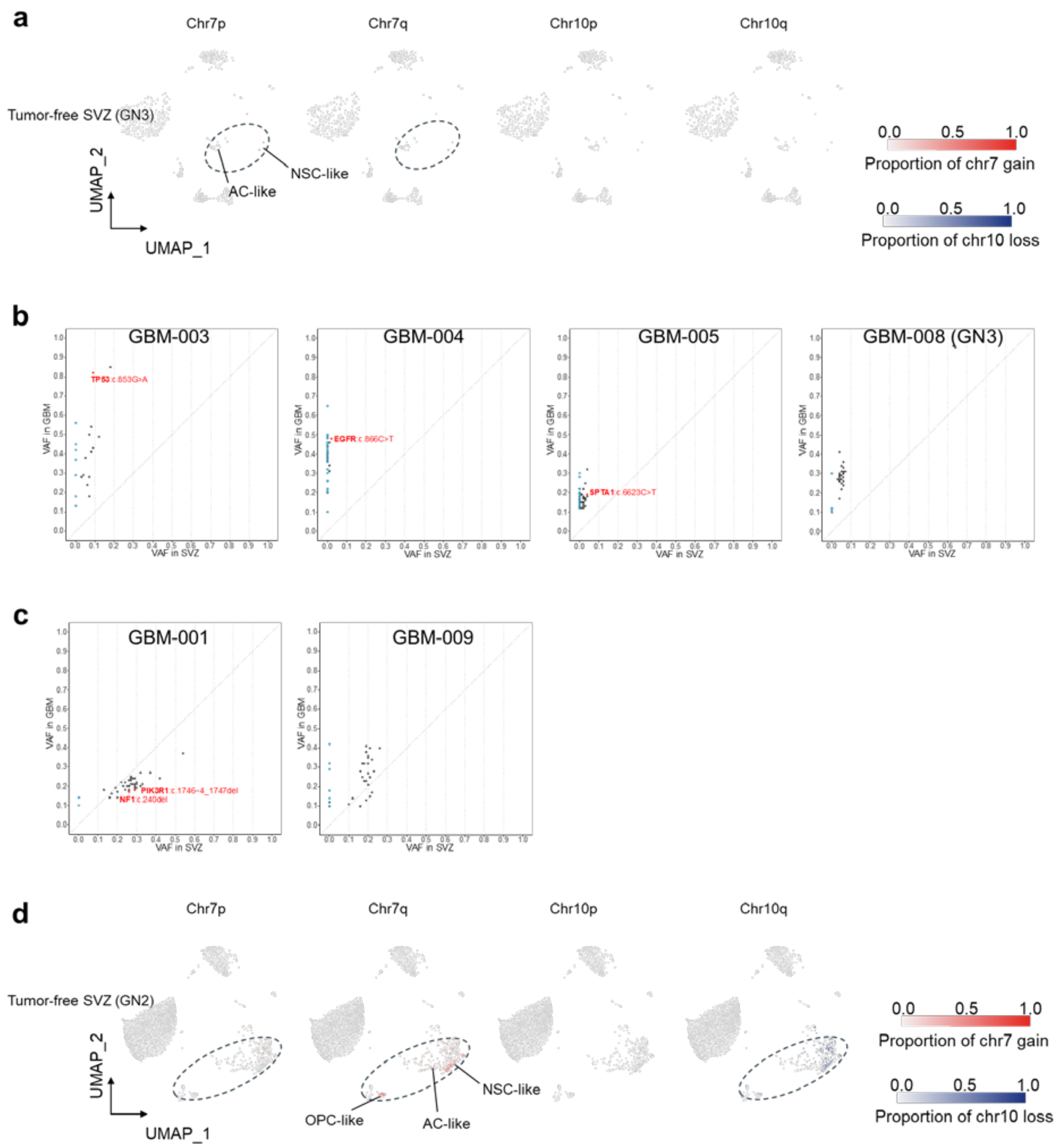

Supplementary Fig. 13 Comparison of snRNA-seq data with somatic mutation analysis in GBM. a UMAP plots depicting chromosome 7p or 7q arm gains and chromosome 10p or 10q arm losses in the tumor-free SVZ of patient GN3 (Left to right: chr7p gain, chr7q gain, chr10p loss, and chr10q loss.). Red and blue color scales indicate inferCNV-inferred, scaled copy-number signals for chromosome 7 gain and chromosome 10 loss, respectively. Dashed circles highlight the CNA-positive region in the tumor-free SVZ. AC-like, astrocyte-like; NSC-like, neural stem cell-like. **b** Scatter plots of mutation variant allele frequencies (VAFs) inferred from patient-matched tumor-free SVZ and GBM samples. High-confidence somatic mutations called in the paired GBM sample were evaluated in the matched tumor-free SVZ (GBM-003,

-004, -005, and -008). The x-axis represents VAFs inferred at the selected GBM mutation sites from tumor-free SVZ, and the y-axis represents VAFs from the corresponding patient-matched GBM. GBM-private mutations are highlighted in blue. **c** Same as **b**, but for paired tumor-infiltrated SVZ and GBM samples (GBM-001 and GBM-009). **d** UMAP plots illustrating chromosome 7p or 7q arm gains and chromosome 10p or 10q arm losses in the tumor-free SVZ of patient GN2. Colors indicate the scaled extent of chromosome 7 gain or chromosome 10 loss. Circles highlight chromosomal alterations detected in the tumor-free SVZ. AC-like, astrocyte-like; NSC-like, neural stem cell-like; OPC-like, oligodendrocyte precursor cell-like.
